# Multifunctional Composites for Energy Storage: Current Trends and Future Perspectives

**DOI:** 10.3390/ma18225168

**Published:** 2025-11-13

**Authors:** Jacek Rduch, Wojciech Skarka, Elena Pastor, Arun Winglin Amaladoss

**Affiliations:** 1 Department of Fundamentals of Machinery Design, Faculty of Mechanical Engineering, Silesian University of Technology, Akademicka 2A, 44-100 Gliwice, Poland; jacek.rduch@polsl.pl (J.R.); arun.winglin.amaladoss@polsl.pl (A.W.A.); 2 Departamento de Química, Instituto de Materiales y Nanotecnología, Universidad de La Laguna, AP 456, 38206 San Cristobal de La Laguna, Tenerife, Spain; epastor@ull.edu.es

**Keywords:** multifunctional, composite, structural, energy storage

## Abstract

Electricity is currently essential for the operation of most modern devices, with significant electrification being observed in all areas. This development has led to an increased demand for solutions that enable energy storage appropriate for a given application, which is currently solved by installing batteries with adequate capacity. This article presents an approach utilizing composite materials that combine both structural and energy storage features. The most frequently discussed components of such materials in the literature are compared, divided into those that contribute to the structural functions of the composite and those that provide additional functionality. The methodology for developing our literature analysis and for comparing materials is given. The results of our publication analysis are then presented, based on the type of integration of multifunctional elements, structural materials, resins, electrolytes, and production methods. The influence of these parameters on the mechanical and electrochemical properties of multifunctional composites is examined. The different materials are compared, and the best ones selected based on appropriate criteria. The current state of knowledge regarding simulations of such materials is presented, and the potential applications of multifunctional composites are reviewed. Finally, key research gaps are identified, suggesting directions for future work.

## 1. Introduction

The classic approach to powering vehicles and devices most often involves installing appropriately sized lithium-ion or lead-acid batteries. Various solutions have a number of advantages and disadvantages, including space requirements and vulnerability to damage, while power density, although constantly being improved, remains a significant limitation. Modern solutions typically strive for higher power densities. An approach that minimizes the space requirement is also crucial. It is important to consider the application in question, as batteries used in critical infrastructure buildings, mobile devices, and vehicles face different requirements, depending on their intended use [1,2].

Another approach involves materials that integrate multiple functionalities, often utilizing existing structural elements for energy storage by employing multifunctional composites.

Currently, for electrical energy storage, three main approaches are most commonly proposed: a lithium-ion cell embedded within a composite (usually a sandwich-type cell), a thin-film cell embedded within the composite, and a structural battery, which often functions as a capacitor. Depending on the solution, this results in a weight reduction of at least 5–10% when replacing a conventional battery with carbon fiber. An additional advantage of using multifunctional composites is the distribution of the battery’s concentrated mass over a larger surface area, which can be very valuable in certain applications, such as aviation. In the case of modern passenger aircraft, such as the Boeing 787, composite materials constitute over 50% of the aircraft’s total mass (80% of its volume). In modern electric cars, the battery system accounts for up to 25% of the vehicle’s weight. The use of multifunctional composites will allow for better space utilization. Eliminating concentrated mass will simplify aircraft design—there will be no need to balance their mass to achieve an optimal center of gravity [3,4,5].

Safety and, consequently, mechanical strength are important factors. The use of composite materials increases the mechanical strength of batteries, allowing the use of such structural batteries without the need for a dedicated space [6,7].

Multifunctional composites allow for the combination of various functions. Photovoltaic panels and piezoelectric materials represent examples of approaches for integrating energy harvesting functionalities with composite batteries. Such integration is complementary, as it combines different methods of energy generation and storage to enhance overall system performance. These components are commonly used individually, but a few attempts have been made to integrate multiple functions [8].

While simulating the behavior of composite materials is not as challenging as electrical simulations of batteries, accounting for the electrical, chemical, and mechanical relationships is a significant challenge. This complicates the practical application of such materials [9].

The increasing demand for multifunctional composite materials capable of electrical energy storage is becoming particularly significant in the fields of general aviation and unmanned aerial vehicles (UAVs). This trend is driven by the ongoing electrification of smaller aircraft, where weight reduction and space efficiency are crucial. Given our expertise in drone technology and general aviation systems, this review focuses on the development and application of multifunctional composites for electrical energy storage, addressing the pressing needs of these rapidly evolving sectors. This article presents a comprehensive overview of studies published between 2015 and 2025, with a focus on multifunctional electric energy storage composites. A thorough analysis of the mechanical and electrical parameters of the tested materials was conducted, revealing the relationships between the components used in production and the composite’s properties. Materials from various publications were compared in terms of their multifunctionality. Among the compared materials, six exhibiting the highest performance parameters were selected for detailed analysis.

### 1.1. Composite Materials

Composite materials consist of two or more different materials. Unlike mixtures, composites can be clearly identified by the appearance of their components. The most commonly used are fiber composites, in which the reinforcement is made of various types of fibers, often forming mats and resin matrices, allowing for the utilization of the advantages of both components. The matrix provides shape and stiffness, while the fibers possess very high tensile strength. The resulting materials are characterized by excellent strength at a relatively low density, benefiting weight reduction [10].

In addition to the materials used, the parameters of a composite depend on its production method and structure. A characteristic feature of composite materials is anisotropy resulting from the orientation of the reinforcement fibers. Adhesion between components also has a significant impact on strength. This adhesion is a result of both the resins used and the production method and conditions [10]. The most commonly used materials for composite construction are described in Section 1.1.1, Section 1.1.2, Section 1.1.3, Section 1.1.4 and Section 1.1.5.

When designing structures made from composite materials, the much more difficult simulation of such materials is a challenge. While homogenization can be performed in some cases, to obtain accurate results, their anisotropy and adhesion must be taken into account [11].

#### 1.1.1. Carbon Fiber Reinforced Polymer (CFRP)

T300 carbon fibers are most commonly used for the production of multifunctional composites [2]. This material is characterized by a nominal Young’s modulus of 294 GPa, a tensile strength of 5490 MPa, and a density of 1.81 g/cm^3^. In combination with 7901 resin and 62% filler, it allows a modulus of 137.7 GPa and a strength of 1784.9 MPa for the force applied in the fiber direction to be achieved [12]. For components requiring higher stiffness, UMS40 or UMS45 fibers are used, characterized by a high Young’s modulus of 395 GPa and 430 GPa, respectively [9]. T800 fiber can meet high tensile requirements, having a tensile strength of 5.49 GPa [13,14].

#### 1.1.2. Glass Fiber Reinforced Polymer (GFRP)

Glass fiber has poorer mechanical properties than carbon fiber, with a typical tensile strength of 0.48–4.58 GPa and a Young’s modulus of 35–86 GPa [10]. It is less frequently used in the production of advanced composite multifunctional materials requiring high strength at low weight. A major advantage, allowing for wider use of these materials, is their price, which is half that of carbon fiber. The most commonly used are E-Glass mats [15,16,17].

#### 1.1.3. Aramid Fiber Reinforced Polymer (AFRP)

Aramid fibers are commonly known as Kevlar. They are characterized by excellent mechanical properties, having a lower density than carbon fibers with similar tensile strength, but with significantly higher elongation at break. An important feature for their use in energy-storing components is that they do not conduct electricity, so can be used as separators in batteries [10,18].

#### 1.1.4. Adhesive Materials

Epoxy resins constitute the most important group of thermosetting adhesive materials used in materials science. Due to their high adhesion to various substrates, excellent mechanical and chemical properties, and low shrinkage during curing, they are widely used in many advanced structural applications. Commercially available composites mostly use resins derived from the diglycidyl ether of bisphenol A (DGEBA resins), the most popular of which is resin 105. The epoxy adhesive E-120HP is often used to bond the core to the outer plates [2,19].

#### 1.1.5. Core Materials

Typical core materials used in sandwich composites are PVC and SAN polymer foam. For cores with a specific structure, ABS and aluminum are used. The most popular structure is a honeycomb, which allows for a four-fold reduction in density while halving the Young’s modulus [2].

### 1.2. Multifunctionality

Multifunctionality is considered whenever a single component is intended to fulfill more than one function. Materials typically combine load-bearing functions with other system functions, such as energy storage, thermal control, or electronics. Integrating these subsystems within a single structure allows for a reduction in the mass and volume of the entire system, which is crucial in many applications [20]. The most frequently integrated components are described in Section 1.2.1, Section 1.2.2, Section 1.2.3, Section 1.2.4 and Section 1.2.5.

#### 1.2.1. Lithium–Polymer (LiPo) Battery

**Figure 1:**
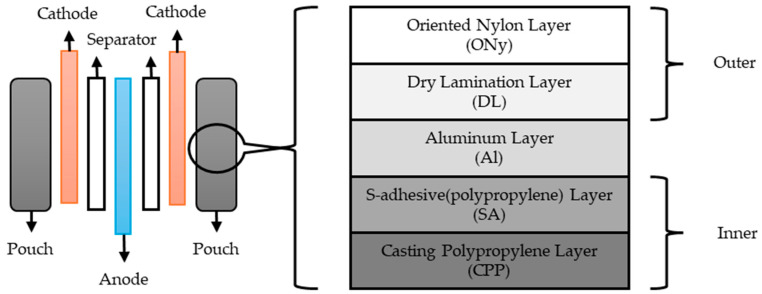
The structure of the LiPo battery and the individual layers of the protective pocket.

In these batteries, the anode and cathode layers are separated by a polymer membrane separator (Figure 1). In the most commonly used batteries, the electrodes are made of copper and aluminum [5,21].

One of the most commonly used batteries is the 3.7 V, 500 mAh battery. These have a volumetric energy density of approximately 385–1286 Wh/L and a mass density of 185–250 Wh/kg. Their use in thin laminates is hampered by their thickness of up to 4 mm [22,23,24,25]. The mechanical properties of such a battery have been experimentally determined [26]. Smaller batteries with a capacity of 50 mAh, which are 0.8 mm thick, are also used [27,28].

Puncture resistance represents a critical property for battery safety and durability. Standard puncture testing showed that dynamic impacts at speeds up to 5 m/s with a 12.7 mm diameter hemispherical plunger and a force of up to 120 kN caused puncture of the battery. Increasing the velocity reduced the force required to short circuit the battery. At the moment of impact, the voltage dropped before the maximum force was reached, although a puncture could occur without a complete voltage drop [29]. Based on the impact at low speeds (up to 500 mm/s), the battery behavior at higher speeds could be successfully predicted [30].

In terms of mechanical strength, these batteries are very sensitive to bending. They are easily deformed, resulting in a loss of electrical properties [5]. During compression, stresses of 4.1 MPa caused plastic deformation, which has no effect on electrical properties [31].

#### 1.2.2. Thin Film Lithium Battery (TFLB)

**Figure 2:**
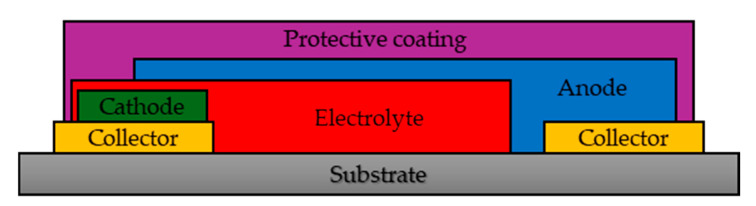
Structure of a TFLB.

Batteries are also produced in the form of foils with a thickness usually below 500 nm. Figure 2 shows the typical structure of a TFLB [32]. Compared to LiPo batteries, their major advantage is their flexibility, which allows for easy adaptation to complex shapes without affecting their electrical properties [33,34].

The use of a solid polymer electrolyte improves operational safety. The use of ethylene oxide and graphene in its construction allowed for a voltage of 4.9 V, a surface capacity of 0.13 mAh/cm^2^, and a volumetric energy density of 4.8 mWh/cm^3^ to be obtained [33]. All Solid State Batteries (ASSB) are characterized by higher mass power density, higher specific power compared to batteries with a liquid electrolyte, and very high possible C-factor values (above 100C, compared to less than 10C for LiPo batteries) [35]. The energy density depends largely on the materials used and can reach up to 320 Wh/kg [36].

#### 1.2.3. Structural Battery

**Figure 3:**
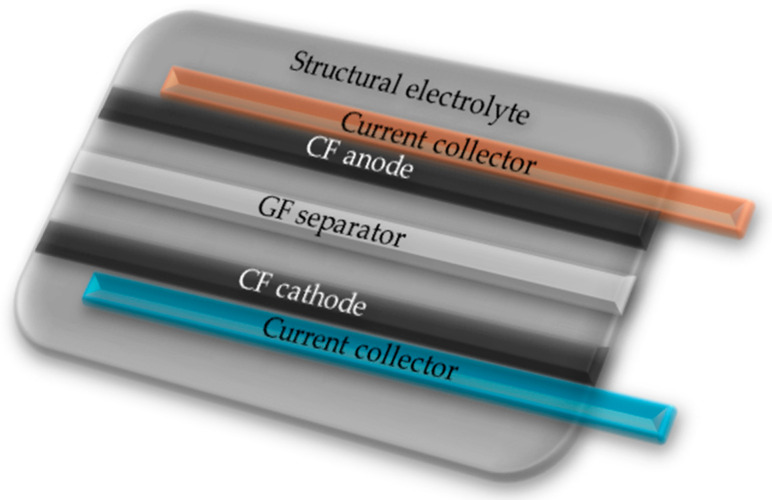
Structure of a structural battery.

Structural batteries (Figure 3) are modern composite materials that combine two functions: mechanical (i.e., load-bearing capacity and structural strength) and electrochemical (i.e., the ability to store and transmit electrical energy). Instead of treating the power source as a separate component, the entire structure (for example, a drone wing, a phone case, or a vehicle component) becomes a battery. This is a particularly attractive direction for applications requiring weight reduction, such as in aviation, spaceflight, and electromobility [37].

The concept is based on the use of carbon fiber composite materials, which are not only lightweight and durable but also conduct electricity well. In solutions proposed in the literature, the battery components were replaced with composite elements. The electrodes are based on carbon fibers, most often enhanced with graphite or lithium. The separator in most materials is glass fiber, less commonly aramid fibers or polymers. The electrolyte, in the form of a liquid, gel, or solid, contains lithium salts and a polymer base. One of the main challenges in the development of structured batteries is the low ionic conductivity of the solid and semi-solid electrolytes used. To address this, researchers are experimenting with electrolytes with optimized structures. One groundbreaking project utilized carbon fibers and graphite as the anode, LiCoO_2_ as the cathode, and a polymer electrolyte (PVDF-HFP-based) enriched with a LiPF_6_ salt. Despite limited ionic conductivity, promising mechanical properties and a decent capacity were achieved. Structural batteries fabricated using additive technologies similar to 3D printing are a further development of this idea, where individual carbon fibers (e.g., IMS65) are coated with a thin layer of a solid polymer electrolyte containing salts such as LiTf. Further research is focused on modifying the fibers, matrix, and composite interfaces to improve both the mechanical and electrochemical functions, making these materials particularly attractive for applications in aerospace, automotive, and portable electronics [2,3,37,38,39].

#### 1.2.4. Integrated Energy Generating Components

Composites can integrate electrical energy-generating components. Photovoltaic panels are most commonly used for energy generation. Their integration as an outer layer of the composite has been tested and used to power a simple IoT device [8].

Piezoelectric materials that convert forces into electrical energy are less commonly used. In some cases, for example, an aircraft wing, they can yield very good results. Simulations have shown that such elements installed in an unmanned aircraft wing can generate 2.31 × 10^−4^ J/s. A combination with a thin-film battery has also been proposed. In addition to being a structural element, such a composite acts as both an energy storage device and a generator [40]. Simulations indicate that the energy that can be generated in wings with a span of 14.5 m can reach 25.24 kW [41].

#### 1.2.5. Integrated Electronics

Another element that can be integrated is an electronic system. These could include sensors for monitoring composite integrity or a thermal management system, which has been identified as a crucial element of energy-storing composites. Health monitoring is equally important, allowing for continuous assessment of the structure’s health. Embedding entire systems or individual components in multifunctional composites has been partially tested. An antenna was successfully integrated within the honeycomb core of a sandwich composite, allowing for a five-fold reduction in its weight while ensuring the required mechanical strength [20].

### 1.3. Production Methods for Multifunctional Composites

This review focuses on composites designed for electrical energy storage. The following sections describe the manufacturing techniques reported in the analyzed literature. The production methods primarily depend on the degree of battery integration, including chemical integration, as seen in structural batteries. Secondly, the components and their resistance to certain factors, such as temperature, must be considered.

Hand lay-up is the classic fiber production method, which involves layering fabrics, pouring resin, and curing at the appropriate temperature [42]. High temperatures should not be used to produce carbon fiber samples with embedded LiPo batteries, as they can damage them. Curing at 20–25 °C for up to 24 h is often used [23,43]. Curing can be extended with an additional cycle at an elevated temperature of 60–65 °C for another 6–12 h. During curing, the material is often put under reduced pressure using a vacuum pump, with pressures in the range of 0.5–1 atm being most commonly used. Curing in a single cycle at elevated temperature is rarely used [2,44,45].

Vacuum Assisted Resin Transfer Molding (VARTM) is a modification of the resin transfer molding (RTM) process, which is widely used, especially in the production of large composite structures. It enables precise control of the fiber-to-resin ratio, ensures process repeatability, and has relatively low manufacturing costs. The VARTM process involves several stages. After cleaning the mold, a release agent and gelcoat are applied. Dry reinforcement layers (e.g., fiber mats) are then laid down, followed by a flow medium. The entire assembly is covered with a flexible vacuum bag and sealed with tape. The resulting vacuum allows the resin to be drawn into the system, where the material is saturated and subsequently cured at room or elevated temperature. The process requires careful design of the feed and vent lines to ensure complete wetting of the structure and avoid void formation [46].

Electrospinning is a method that yields very good results for integrated batteries. It has been proven that samples made with this method have better properties than similar samples made using the air-spray method, which in turn have better properties than those made conventionally. The use of electrospinning allows a battery’s properties to be preserved under mechanical stress. To produce a sample with an embedded LiPo battery, a carbon fiber prepreg was used, arranged in 16 layers, of which the central four were cut out to make room for the battery. Curing was performed using a vacuum pump for 60 min at room temperature, followed by 25 min at 120 °C and a pressure below 1 bar. After this time, the composite was cooled while maintaining the reduced pressure. The battery inside the composite was filled with resin, which was cured at 100 °C and a pressure of 0.5 MPa. The resulting composite had a thickness of 5.2 mm [5,27].

3D printing solutions can be successfully applied to carbon and glass fiber composites. The FDM method allows for rapid prototyping without the need for expensive molds. Continuous fiber methods yield particularly good results. Structural battery layers can also be produced using the drop-on-demand method, which is similar to 3D printing. This allows for the addition of various solutions to individual layers. Based on tests, the optimal layer height was determined to be 0.4 mm, which corresponded to a 35% volume fill with carbon fiber [16,47].

## 2. Methodology

This review addresses multifunctional composites for electrical energy storage, with a focus on applications in general aviation and UAVs. Studies from 2015 to 2025 were selected based on their relevance to combining mechanical performance with energy storage functionality. The methodology involves systematic comparison and analysis to highlight key material-property relationships relevant to lightweight electrified aircraft.

### 2.1. Literature Analysis

The Google Scholar and Scopus databases were analyzed for titles, abstracts, and keywords. Articles were searched for using terms such as “multifunctional energy storage composite,” “thin film integrated composite,” “lithium polymer integrated composite,” “structural battery,” “composite with embedded lithium-ion batteries,” and “multifunctional structural battery.” To select only high-quality publications, attention was paid to the number of citations, discarding publications with none. For articles published in 2024–2025, the lead author’s work and H-index were considered, as these publications were deemed too recent to receive citations.

To characterize the materials and compare them, a list of parameters was compiled, taking into account their applications as structural and energy storage materials. Properties were categorized based on their function as [3,9]:

- Electrochemical for energy storage elements:
  - Mass energy density [Wh/kg]
  - Volume energy density [Wh/L]
  - Unit capacity [F/g]
  - Dielectric strength (number of charge/discharge cycles)
- Mechanical for structural elements:
  - Elastic modulus (Young’s modulus) [GPa]
  - Compression modulus (Helmholtz modulus) [GPa]
  - Bending modulus (Bending modulus) [GPa]
  - Shear modulus (Kirchhoff modulus)
  - Tensile strength [MPa]
  - Compressive strength [MPa]
  - Bending strength [MPa]
  - Shear strength [MPa]

In their publications, authors usually report capacity in mAh/g, which cannot be directly compared between energy storage devices with different voltages. Therefore, the capacitance C in F/g was determined as the amount of stored charge according to Equation (1). This approach takes the battery voltage into account and allows for comparison between materials.

(1) $$C=\frac{{Q\cdot (V}_{max}+V_{min})}{V_{max}^{2}-V_{min}^{2}}$$

where

*V_max_*—Upper voltage limit

*V_min_*—Lower voltage limit

*Q*—Specific charge [C/g] determined according to Equation (2)

(2) $$Q=C_{mAh/g}\cdot 3.6$$

The testing method for determining the above properties is specified in standards. It should be noted that the results obtained using one method may differ from those obtained using other methods. In mechanical testing, force may be applied slowly, resulting in quasistatic tests, or at a higher rate, resulting in dynamic tests. Fatigue testing constitutes a separate category. During these tests, the material is subjected to cyclic loading, determining the material’s service life, which is particularly important in applications where fatigue strength affects safety, such as aviation or automobiles. The most commonly used test methods for determining material properties are:

- Electrical and electrochemical:
  - Charge–discharge cycles with a defined C-factor, typically 1, corresponding to a current draw sufficient to discharge the battery in one hour [5]
  - Tests according to ISO12405-4 [48]
  - Impedance measurement [25]
- Mechanical:
  - Three-point bending per ISO 14125: quasistatic, for a specified time [5,49,50]
  - Three-point bending per ASTM D790 [51]
  - Shear test [26]
  - Uniaxial compression [26]
  - Low-velocity impact [24]
  - Dynamic impact (up to 5 m/s) [29]
  - Thermal vacuum testing [52]

### 2.2. Comparison of Materials

Composite materials can be classified based on the degree of integration of the battery with the structural element:

- Integrated battery—the battery is embedded as a separate component within the composite. This group includes:
  - LiPo (pouch) batteries—most commonly used in sandwich composites.
  - Thin-film batteries—batteries of very thin thickness, capable of being inserted between fiber layers.
- Structural battery—structural elements, such as carbon fiber, are also components of the battery. The battery itself cannot be clearly distinguished.

To determine the quality of a multifunctional composite, the concept of efficiency was introduced, which provides information on whether the developed multifunctional material is better than the monofunctional materials it contains. It was defined as the sum of the efficiency of individual functions [3,9]:

(3) $$n_{mf}=n_{e}+n_{s}$$

where

$n_{e}$—Electrochemical efficiency

$n_{s}$—Structural efficiency

These values can be calculated by relating a given size of the multifunctional material to the corresponding property of the component:

(4) $$n_{n}=\frac{X_{mf}}{X}$$

where

$n_{n}$—Efficiency with respect to a given property

$X_{mf}$—Property of the multifunctional material

X—Property of the reference element

The resulting efficiency of n_mf_ > 1 means that a given composite material offers greater benefits than the individual components it comprises. Therefore, a reduction in the mass of the system in which it is used and replaces the individual components can be expected [3].

It is important to properly select the properties being compared, depending on the intended use of the analyzed material. Similarly, more properties can be compared using the minimum criterion:

(5) $$n_{n}=min\left\{\frac{{X1}_{mf}}{X1},\frac{{X2}_{mf}}{X2},\ldots ,\frac{{Xj}_{mf}}{Xj}\right\}$$

**Figure 4:**
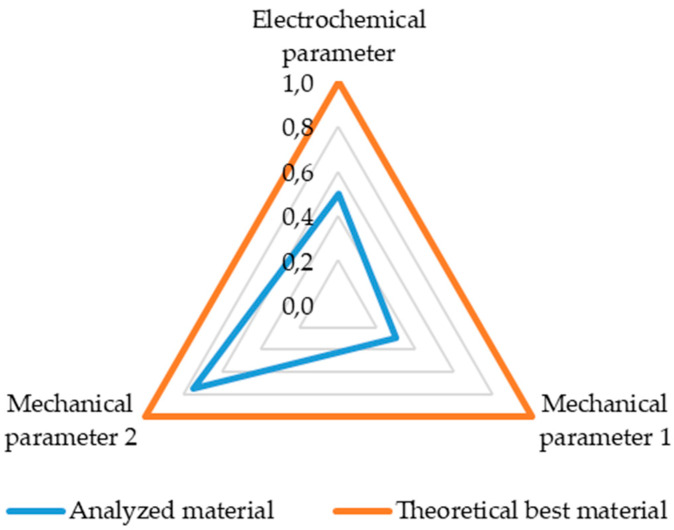
Radar chart example.

To compare materials with each other, due to the unknown parameters of the reference materials, which prevent the determination of multifunctionality, a different method is necessary. A methodology was developed to compare the analyzed materials. For this purpose, key parameters were selected for comparison. Three parameters were compared simultaneously, including one corresponding to the energy storage functionality and two corresponding to the structural element. It should be noted that Figure 4 is intended as a conceptual example illustrating the methodology of comparing materials, rather than presenting actual experimental data. These values were normalized across all analyzed materials for which a given value was known and then presented in a radar chart (Figure 4). The normalization procedure was performed using a min–max approach, where each value $x_{i}$ for a given parameter was normalized according to (6). In cases where a material did not report a value for a specific parameter, it was excluded from the normalization for that particular comparison, ensuring that only available and reliable data were considered in the radar charts and subsequent comparative analyses.

(6) $$x_{\mathrm{norm}}=\frac{x_{i}-x_{min}}{x_{max}-x_{min}}$$

The surface areas for each material were then calculated and compared to the maximum possible value of 1.3 (theoretical best material). In the example presented above, the surface area of the analyzed material is 0.325, which corresponds to 25% of the maximum area. This approach allows visualization of the trade-offs between different properties and demonstrates how the methodology can be applied to evaluate multifunctionality, even though not all properties are directly comparable across materials. The resulting areas determine which material is better, taking into account all three characteristics.

## 3. Literature Analysis Results

**Figure 5:**
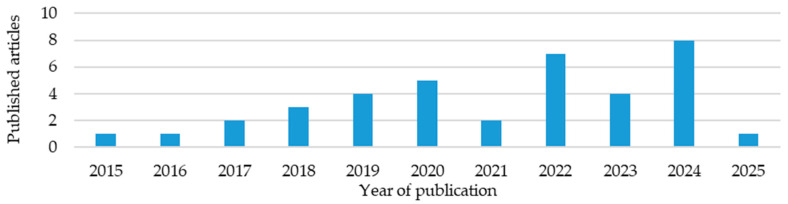
Number of analyzed articles published in each year [5,7,8,22,23,24,25,27,31,42,44,45,47,49,52,53,54,55,56,57,58,59,60,61,62,63,64,65,66,67,68,69,70,71,72,73,74,75,76].

This review analyzed 67 different composite multifunctional materials. In the graphs and tables, only references to the source publications are provided. Detailed descriptions and analyses of selected studies are presented at the end of the review during the comparative analysis, to maintain the clarity and readability of the presented data. A growing interest in such materials is evident (Figure 5). Many articles from 2025 had not yet been published at the time of this review. The articles were then grouped by integration type, materials used, resins used, and, in the case of structured batteries, additionally by electrolyte. The methods and conditions for producing these materials were also analyzed.

### 3.1. Proposed Approaches for Multifunctional Electric Energy Storage Composites

The following chapters describe the approaches proposed in the literature according to the classification introduced in the methodology: integrated LiPo battery, integrated TFLB, and structural battery.

#### 3.1.1. Laminate and Sandwich Composite with an Integrated LiPo Battery

**Figure 6:**
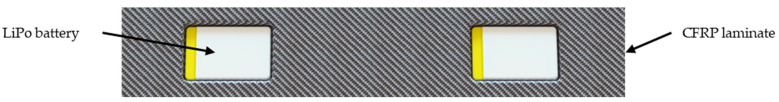
Lithium polymer batteries embedded in CFRP.

This is a solution frequently used in the literature. It provides a small weight reduction with an energy density of 20–139 Wh/kg (20–276 Wh/L). The widespread availability and diversity of LiPo batteries contribute to their cost-effectiveness, but the manufacturing process, which requires the preparation of cutouts for battery placement, poses a problem [9]. Foams are most often used as the core surrounding the embedded batteries. In sandwich composites with a honeycomb core, inserting a LiPo battery did not significantly affect the electrical and mechanical properties. The electrical properties were maintained up to 60% of the breaking load during three-point bending; after this value, a steady capacity decrease occurred [25]. In the case of a low C-factor, bending also had no significant effect on the electrochemical properties [45]. The possibility of manufacturing such materials in the form of a laminate has also been proven (Figure 6), but in this case the strength was always significantly reduced. It is worth noting, however, that the effect of stretching on the electrical properties was negligible [23]. This solution offers the least possibility of shaping the mechanical and electrical parameters of the multifunctional material, as they depend primarily on the batteries used and their quantity [22]. In previous studies, composites of this type have been examined in many respects; often, several systems were analyzed within a single publication due to the ease of obtaining the material and the possibility of using various combinations of battery arrangement [23].

The advantage of using this solution with an appropriate battery arrangement is improved acoustic properties and vibration damping of the material [22], also increasing the amount of energy the material can absorb [24]. An impact energy absorption of up to 80 J was observed without a significant decrease in electrical properties when the impact speed was reduced [44]. A laminated composite can absorb significantly less energy, with a negligible effect on capacity and resistance observed for impacts up to 4 J. An impact energy above 6 J caused deformation and internal short circuits in the battery, and an impact with energy of 10 J resulted in the battery being forced out of the composite.

Discharging batteries, especially those with a high current draw, leads to their heating. Tests conducted at an ambient temperature of 21 °C revealed an increase of 29 °C for a free-standing battery when discharged at a current of 5 A. Embedding in a laminate allowed for a 50% reduction in heating due to the good thermal conductivity of carbon fibers [43].

The literature suggested that the volumetric density of a multifunctional material was 50 Wh/l, which caused a reduction in the elastic properties by 30% compared to the composite without a battery [76].

#### 3.1.2. Laminate Composites with an Integrated TFLB

**Figure 7:**
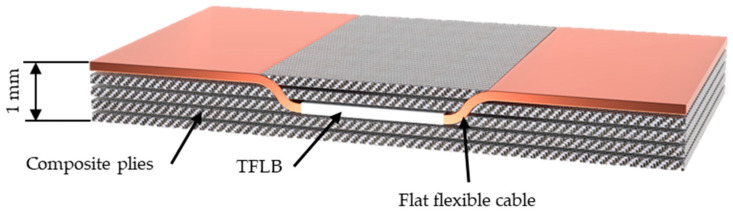
Schematic diagram of a laminate with an integrated TFLB.

A thin battery is placed between the laminate layers (Figure 7) [77]. Its thinness allows for the omission of cutouts in the fibers. These batteries utilize a solid electrolyte, which provides better mechanical properties than batteries with liquid or gel electrolytes. Their flexibility allows for the formation of more complex shapes. Due to their higher price and lower capacity, which hinders scalability, they are rarely selected for multifunctional materials and are therefore much less studied. However, some tests have been conducted, such as three-point bending according to ASTM C393 [78] in a quasistatic manner at a speed of 3.33 m/min, holding in a bent state for 12 h, and subjecting the batteries to 100 discharge cycles at a rate of 1C at 30 °C. These tests confirmed the good mechanical properties of such composites [5].

#### 3.1.3. Integrated Structural Battery

**Figure 8:**
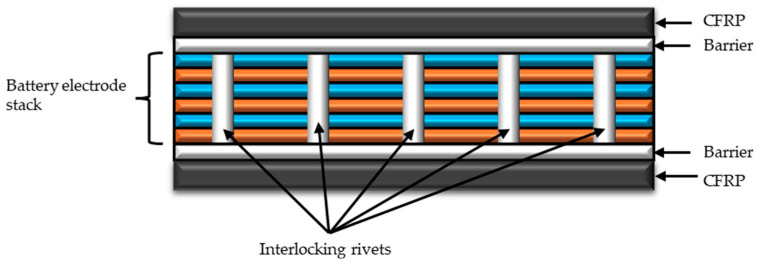
Structural battery reinforced with polymer pins.

A structural battery can be in the form of a laminate, where individual layers form part of the battery, or in the form of a sandwich, where the layers responsible for energy storage are contained within a core. The laminate approach is the most frequently studied. A solution using a structural battery as the core of a sandwich composite has also been tested. It was characterized by high internal resistance and an electrical density of 223 Wh/L. It was proven that the orientation of the layers had a significant impact on the mechanical properties, which could be reduced by using reinforcements in the form of C-shaped sections [79]. Glass or aramid fibers can be used to separate the battery layers. Carbon fiber has been successfully used as electrodes. The electrolyte is most often based on lithium compounds, which gives good results, as described later in the present article. In the developed materials, attempts have also been made to use graphene platelets, resulting in a battery withstanding over 8000 charging cycles [8]. It has also been shown that the use of a solid electrolyte in a structural battery can increase the Young’s modulus of the laminate [58]. Reinforcing composites with polymer pins has also been proposed (Figure 8). In this solution, the surface layers were made of carbon fiber, while the core consisted of anode and cathode layers separated by separators with holes. The pins, passing through the holes and connecting to the surface layers, prevented the composite layers from shifting. This resulted in improved bending strength and the retention of electrical properties through 1000 loading cycles of 700 N. The disadvantage of this solution is reduced energy density [7].

### 3.2. Overview of Statistics by Integration Type

**Figure 9:**
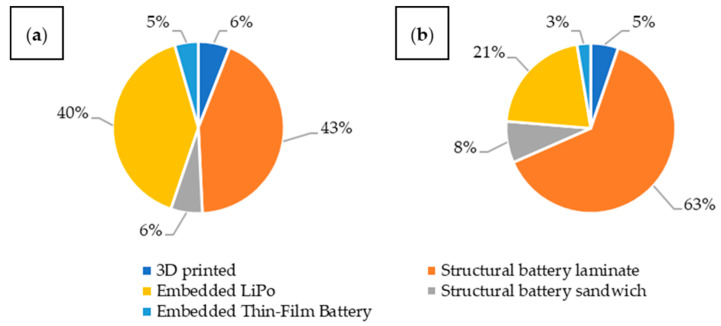
Percentage share of a given integration type in (**a**) Published materials; (**b**) Published articles [5,7,8,22,23,24,25,27,31,42,44,45,47,49,52,53,54,55,56,57,58,59,60,61,62,63,64,65,66,67,68,69,70,71,72,73,74,75,76].

The vast majority of the analyzed articles are structural batteries in the form of laminates (Figure 9a). The second large group of materials are composites with embedded lithium-ion batteries. The situation changes somewhat when considering articles describing a specific type of integration rather than the materials themselves (Figure 9b). This is because publications on composites with embedded batteries often analyzed various configurations and quantities of batteries, resulting in a single article describing several materials. Materials manufactured using 3D printing methods are included in the comparison as a separate type of integration. These, along with composites with embedded lithium-ion batteries in thin-film form, constitute the least popular solutions. Structural batteries in the form of a sandwich composite are slightly more popular. For further analysis, we decided to focus on structural batteries and composites with embedded lithium-ion batteries.

**Figure 10:**
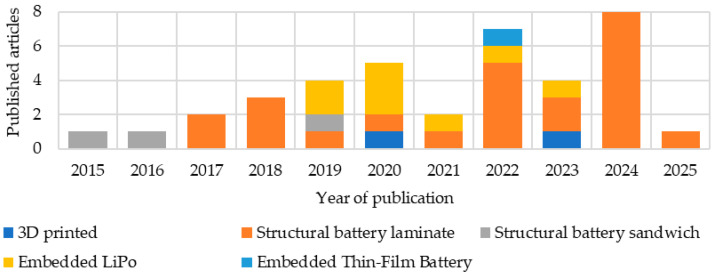
Articles published in successive years with a given type of integration [5,7,8,22,23,24,25,27,31,42,44,45,47,49,52,53,54,55,56,57,58,59,60,61,62,63,64,65,66,67,68,69,70,71,72,73,74,75,76].

Analyzing publications over the years (Figure 10), a growing interest in structural batteries, particularly in the form of laminates, can be observed. Between 2019 and 2021, an increase in publications describing composites with embedded lithium polymer batteries was observed, at the expense of structural batteries.

**Figure 11:**
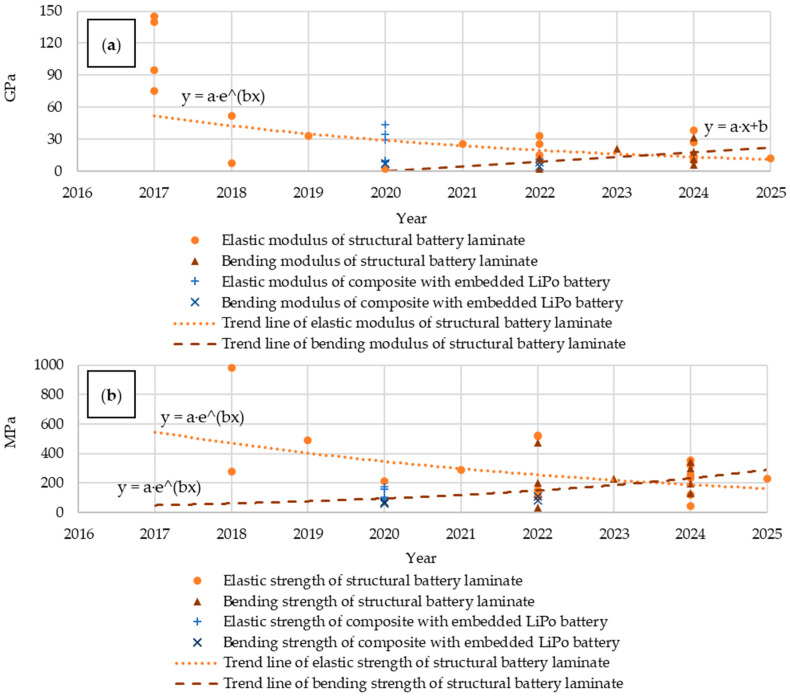
Mechanical properties over successive years for the considered integration types: (**a**) Flexural and elastic modulus; (**b**) Tensile and bending strength [8,23,27,42,54,55,57,58,59,60,62,63,64,65,67,68,69,70,71,72,73,75,76].

Analyzing the mechanical properties, an increasing trend in flexural modulus and flexural strength can be observed, at the expense of decreasing values for Young’s modulus and tensile strength (Figure 11). Prior to 2018, significant emphasis was placed on the high mechanical parameters of structural batteries. In subsequent years, a decline was followed by a renewed increase due to optimization. Structural batteries also demonstrate an advantage over composites with embedded LiPo batteries in terms of tensile and flexural strength, but the number of these materials with their analyzed parameters tested is insufficient to determine trends for the period 2015–2025.

**Figure 12:**
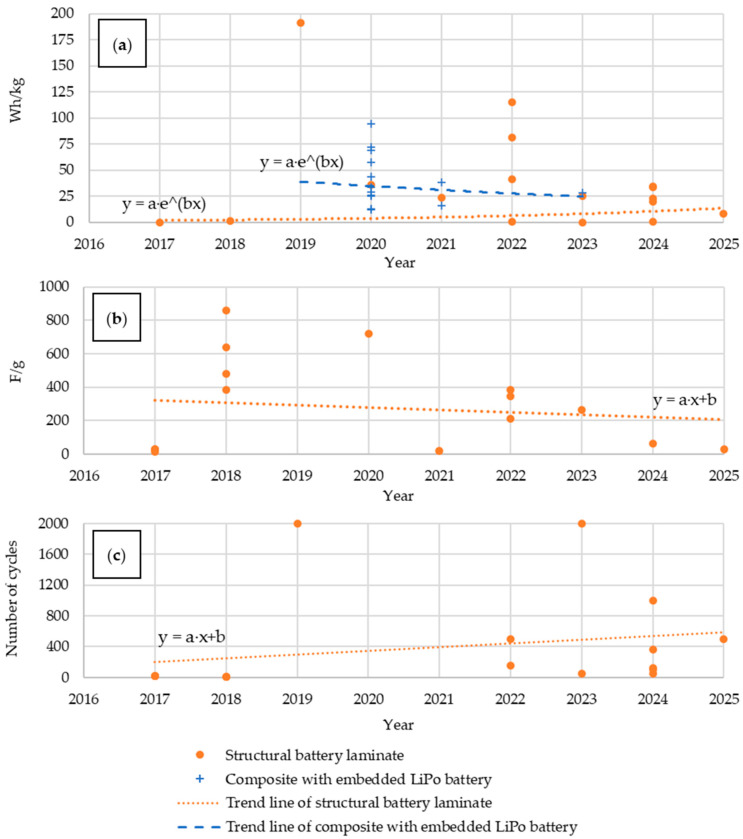
Electrochemical and electrical properties over successive years for the considered integration types: (**a**) Mass energy density, (**b**) Specific capacitance, (**c**) Electric strength [8,23,24,31,42,44,54,55,56,57,58,59,60,61,62,63,64,65,67,68,69,70,71,72,73,74,75,76].

For the analysis of electrochemical properties, we selected mass energy density (Figure 12a), which defines the amount of energy stored per unit mass, specific capacity (Figure 12b), and electric strength, defined as the number of charge and discharge cycles at a C-rate of 0.1 (Figure 12c). The mass energy density for materials with embedded LiPo batteries was higher than for most structural batteries, but it systematically increased in the latter group. Unit capacity, on the other hand, tended to decrease slightly, while the electric strength increased significantly. This trend demonstrates the priority given to strength over electric capacity in previous studies, with strength increasing relatively faster than capacity decreasing.

### 3.3. Review of Statistics Concerning the Applied Structural Material

**Figure 13:**
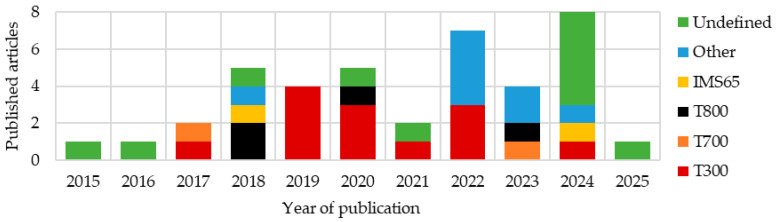
Articles published over successive years with a given type of material [5,7,8,22,23,24,25,27,31,42,44,45,47,52,53,54,55,56,57,58,59,60,61,62,63,64,65,66,67,69,70,71,72,73,74,75,76].

In the analyzed articles, a large group is categorized as “other,” representing materials reported in only a single publication. Among these and other vaguely defined categories, CFRPs still constitute the vast majority. It is also worth noting the significant number of articles in which the material was not clearly and fully specified. An analysis of publications from 2015 to 2025 (Figure 13) shows that such articles make up most of the latest materials. Additionally, a notable number of publications utilizing T300 fibers were published in 2019–2020, coinciding with the peak of research on composites embedded with LiPo batteries.

**Figure 14:**
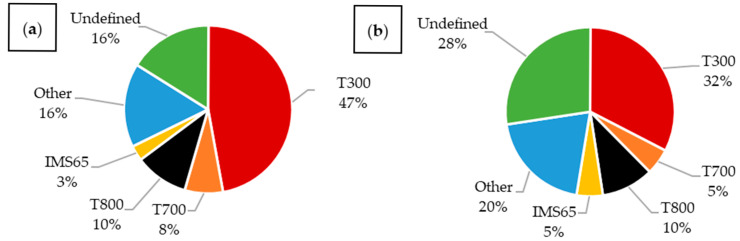
Percentage share of a given material type in (**a**) Published materials; (**b**) Published articles [5,7,8,22,23,24,25,27,31,42,44,45,47,52,53,54,55,56,57,58,59,60,61,62,63,64,65,66,67,69,70,71,72,73,74,75,76].

In the analyzed articles (Figure 14), T300 carbon fibers were by far the most commonly used. These are standard fibers, widely applied in general-purpose constructions [8].

The second largest group of described fibers were T800, characterized by a high tensile strength and Young’s modulus [8].

The next group consisted of T700 fibers, which exhibit significantly higher tensile strength while maintaining a similar modulus to T300 fibers, though they are less strong than T800 fibers [8].

IMS65 materials were used with a frequency comparable to T700. These fibers have an elevated Young’s modulus and tensile strength, similar to T800 [80].

**Figure 15:**
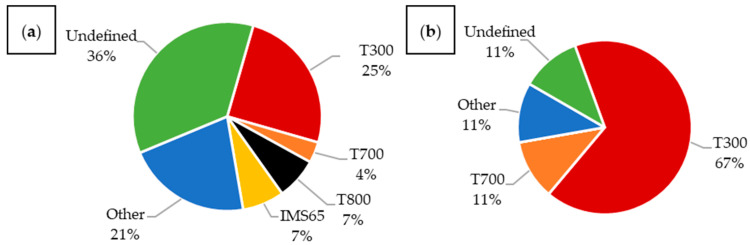
Percentage share of a given material type in (**a**) Structural batteries; (**b**) Composites with embedded LiPo batteries [5,7,8,22,23,24,25,27,31,42,44,45,47,52,53,54,55,56,57,58,59,60,61,62,63,64,65,66,67,69,70,71,72,73,74,75,76].

Analyzing publications that investigated structural batteries (Figure 15a), T300 fibers were the most popular, constituting 25% of all published composites; however, materials were often not specified precisely. Many articles only mentioned the use of carbon fibers without further details. In composites embedded with LiPo batteries (Figure 15b), T300 fibers dominated the majority. The second most common material was T700 fibers. Other types, such as T800 and IMS65, were not used. The large number of materials with imprecisely defined fibers presents a significant challenge for reproducibility and reliable comparison with other materials.

**Figure 16:**
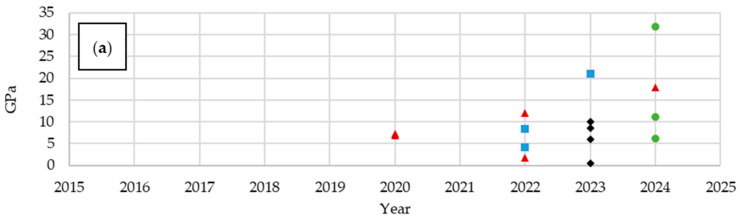

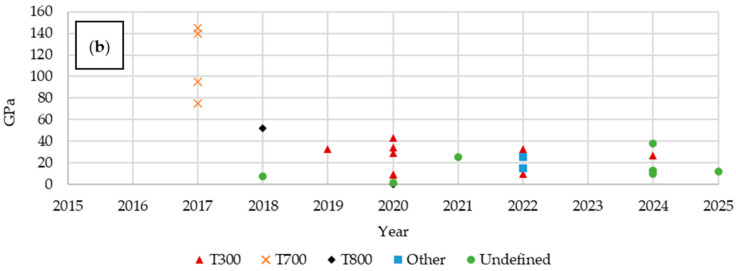
Mechanical properties: (**a**) Flexural modulus; (**b**) Young’s modulus [8,23,27,42,53,54,55,57,58,59,60,62,63,64,65,67,68,70,71,72,73,75,76,80].

Next, the mechanical and electrical properties of multifunctional composites based on the previously described materials were analyzed. At this stage, the distinction between structural batteries and composites with embedded LiPo batteries was omitted in order to obtain a more comprehensive overview of the properties. Over the years, an increase in flexural modulus was observed, accompanied by a decrease in Young’s modulus (Figure 16). The tensile and flexural strengths did not show such distinct trends. The choice of carbon fibers significantly influences mechanical performance: T700 fibers enabled the achievement of high strength parameters, while T300 fibers resulted in the most diverse property values. Notably, T800 fibers exhibit a remarkable potential for producing composites with exceptional mechanical properties; however, their use also led to materials with very low performance in certain cases, highlighting the sensitivity of the composite properties to processing conditions and fiber-matrix interactions. These differences are not only structurally relevant but also provide a foundation for the development of multifunctional materials, where mechanical robustness must be balanced with electrochemical performance. Additionally, the chemical characteristics of the fibers, including surface chemistry and compatibility with the polymer matrix, can affect both the mechanical behavior and potential multifunctional properties, highlighting the interplay between structure, chemistry, and performance.

**Figure 17:**
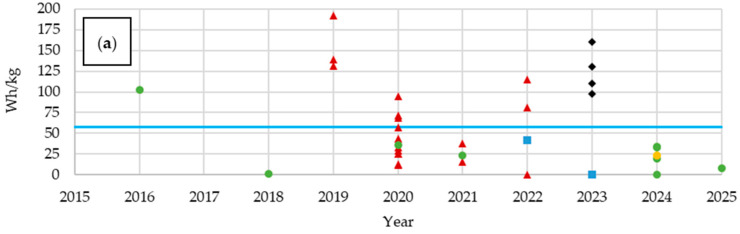

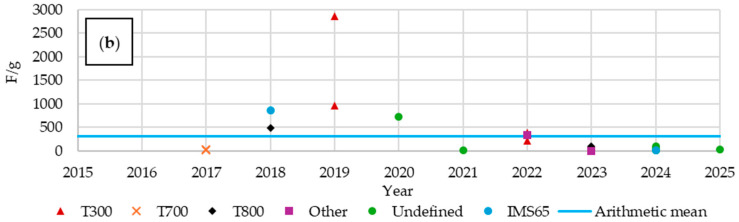
Electrochemical properties: (**a**) Mass energy density; (**b**) Specific capacity [7,8,23,24,25,31,42,45,47,54,55,57,58,59,60,61,62,63,64,65,67,68,69,70,71,72,73,75,76].

In multifunctional composites, mass energy density is an important parameter (Figure 17a). Analyzing the materials used and the corresponding values reveals certain trends. To facilitate comparison, the average value derived from the analyzed publications was marked on the graph. Composites based on T800 fibers consistently exhibited above-average mass energy density values. Articles involving IMS65 fibers or other materials reported composites with values below the average. Unspecified fibers also resulted in below-average values, with the exception of one material from 2016. Once again, T300 carbon fiber showed a wide application range, with both very high and very low values.

Figure 17b presents the specific capacitance (F/g) of the analyzed materials. The limited number of data points reflects the fact that this parameter is rarely reported in the literature. In some cases, authors provided capacity in Ah instead of Farads (F), omitted voltage information, or reported solely energy density, making direct comparisons difficult. Additionally, the diversity of electrochemical systems currently investigated (e.g., lithium-based, iron-based, or polymer electrolytes) resulted in multi-voltage operation ranges, which further complicates direct comparison of capacity data. Therefore, expressing the results in Farads allows normalization with respect to voltage and facilitates a more meaningful evaluation of the performance of multifunctional materials across different chemistries. The use of T800 fibers led to composites with an average unit capacity. T300 fibers again showed a broad distribution of values, both above and below the average. Composites based on T700 fibers and most of those with unspecified reinforcement exhibited below-average specific capacities. One undefined material had a specific capacity above average. Despite the sparse dataset, the observed values highlight specific capacitance as an important property and a potential avenue for further optimization of multifunctional composites.

### 3.4. Statistical Overview Based on the Types of Resins Used

Based on the analyzed materials and the resins used, four groups were identified:

DGEBA—The most commonly used type of epoxy resin in composite materials and protective coatings. It is characterized by high stiffness, good processability, and favorable electrical insulation properties [81]. This group includes all materials based on DGEBA, except for the West System 105 resin, which was treated as a separate group due to its frequent occurrence.

West System 105—Epoxy resins based on DGEBA produced by West System (Bay City, MI, USA). They enable easy wetting and impregnation of fibers. Once cured, they offer high mechanical strength as well as good chemical and thermal resistance [82].

Other—This group includes all other resins that were clearly defined but appeared in only a single publication.

Undefined—This includes materials for which the resin type was not specified, as well as those where the only information provided was the general term “epoxy resin.”

**Figure 18:**
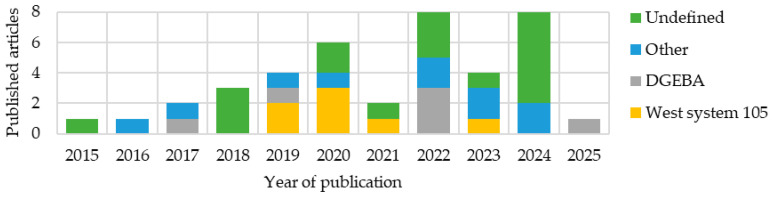
Articles published over successive years by the type of resin used [5,7,8,22,23,24,25,27,31,42,44,45,47,52,53,54,55,56,57,58,59,60,61,62,63,64,65,66,67,68,69,70,71,72,73,74,75,76].

An analysis of publications concerning multifunctional materials (Figure 18) reveals a trend of underestimating the importance of the resin used. There is also a growing tendency to utilize unconventional resins. Such an approach complicates the analysis of the influence of resins on the properties of multifunctional materials.

**Figure 19:**
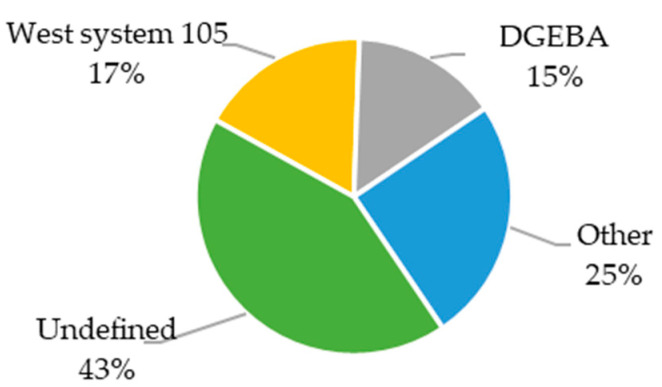
Proportional distribution of resin types across the reviewed publications [5,7,8,22,23,24,25,27,31,42,44,45,47,52,53,54,55,56,57,58,59,60,61,62,63,64,65,66,67,68,69,70,71,72,73,74,75,76].

Among all the published materials (Figure 19), the most frequently reported resins are those from the West System 105 series. However, in most publications, the applied resin was not clearly or precisely specified.

**Figure 20:**
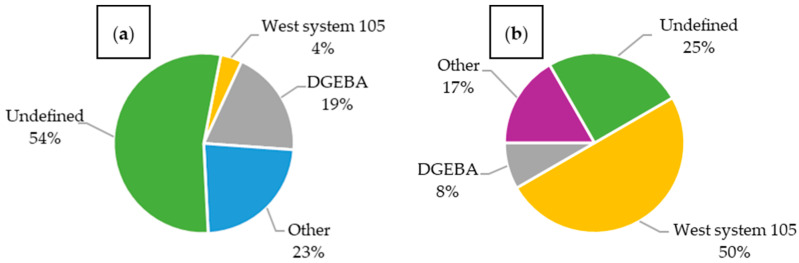
Proportional distribution of resin types in publications related to (**a**) Structural batteries; (**b**) Composites with embedded LiPo batteries [5,7,8,22,23,24,25,27,31,42,44,45,52,54,55,56,57,58,59,60,61,62,63,64,65,66,67,68,69,70,71,72,73,74,75,76].

In the case of structural batteries (Figure 20a), more than half of the articles involved unspecified resins. Among the defined ones, DGEBA-based resins were used most frequently. In composites with embedded LiPo batteries (Figure 20b), the type of resin was specified much more often. The most used was West System 105 resin, while DGEBA accounted for less than 10% of the resins used in this type of composite.

**Figure 21:**
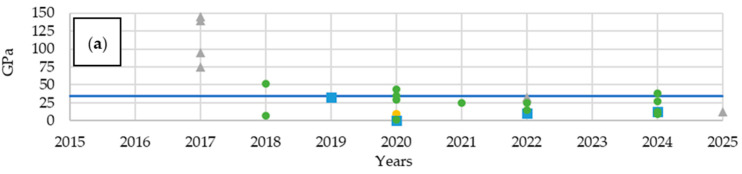

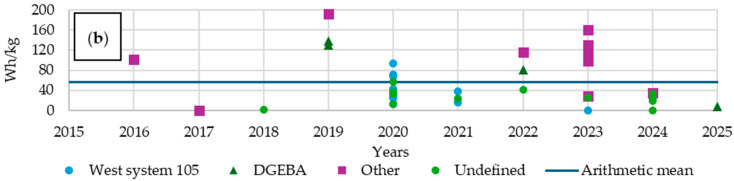
Mechanical and Electrochemical properties: (**a**) Young’s modulus; (**b**) Mass energy density [7,8,23,24,31,42,44,45,47,53,54,55,56,57,58,59,60,62,63,64,65,67,70,71,72,73,74,75,76].

Due to the large number of undefined resins as well as those classified under the “Other” group, analyzing their impact on mechanical properties is very challenging. In the case of Young’s modulus (Figure 21a), undefined resins are positioned around the average value, which is elevated by DGEBA resins. West System 105 resin and other less common resins resulted in materials with a below-average Young’s modulus. On the other hand, DGEBA and other resins mostly enabled above-average mass energy densities to be achieved (Figure 21b). Materials with undefined resins performed the worst in terms of this parameter. Composites with West System resins exhibited values close to the average among the analyzed multifunctional materials.

### 3.5. Review of Statistics Regarding the Electrolytes Used in Structural Batteries

In the case of structural batteries, the electrolyte is a key component responsible for multifunctionality, particularly for electric energy storage. Among all the analyzed materials, the electrolyte was both the most thoroughly described and the most diverse component. Electrolytes identified in at least two publications were classified into nine groups based on their chemical composition. The remaining electrolytes, which appeared only once, were divided into two categories depending on whether or not they contained lithium:

LiTFSI—a lithium salt widely used in lithium-ion battery electrolytes. At room temperature, it appears as a white powder or crystalline solid. It exhibits greater thermal resistance and higher efficiency compared to LiPF_6_ [83].

ZnTFSI—a zinc salt known for its high electrochemical stability, good ionic conductivity, and resistance to decomposition under electrochemical conditions. Due to the presence of the TFSI anion, ZnTFSI demonstrates low reactivity with electrodes and stable performance across a wide potential range [84].

LiPF_6_—a commercially used lithium salt in lithium-ion battery electrolytes. It is characterized by poor thermal and moisture stability, as well as a limited charging rate. The performance of batteries containing this salt is generally considered unsatisfactory, prompting the search for alternatives [83].

EMIM TFSI—an ionic liquid based on the EMIM^+^ and TFSI^−^ ions, characterized by high conductivity and commonly used in gel-based electrolytes [85].

EMIM BF_4_—an ionic liquid with low viscosity, high ionic conductivity, and a wide electrochemical window [86].

LiTf—a lithium salt with high ionic conductivity and excellent chemical, electrochemical, and thermal stability. It is often used in lithium battery electrolytes, particularly in combination with polymers that have low solvation capacity [87].

Na—this group includes electrolytes containing sodium sulfate (Na_2_SO_4_), sodium perchlorate (NaClO_4_), and NASICON (such as Na_3_Zr_2_Si_2_PO_12_).

LiClO_4_—offers good solubility, a wide electrochemical stability window, and high resistance to hydrolytic decomposition. Its major drawback is the significant explosion risk [83].

Undefined—includes electrolytes that were not precisely described in the respective publications.

Other lithium-based—electrolytes that appeared only once and contain lithium compounds.

Other—all remaining electrolytes.

**Figure 22:**
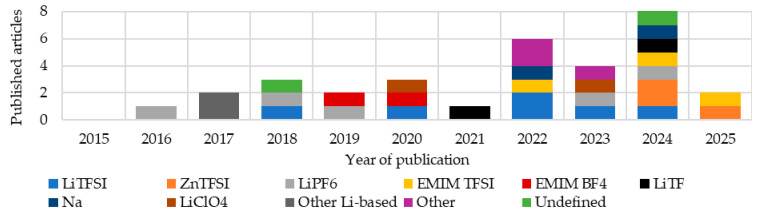
Articles published over successive years by the type of electrolyte used [7,8,42,45,47,53,55,56,57,58,59,60,61,62,63,64,65,66,67,68,69,70,71,72,73,74,75].

An analysis of the changes in the materials used between 2015 and 2025 (Figure 22) revealed growing interest in non-lithium-based electrolytes, particularly in 2024–2025. Nevertheless, lithium remained a component in over half of the structural batteries developed. Another compound frequently present in both lithium-based and lithium-free electrolytes was TFSI, which appeared in more than 37% of the studied batteries.

**Figure 23:**
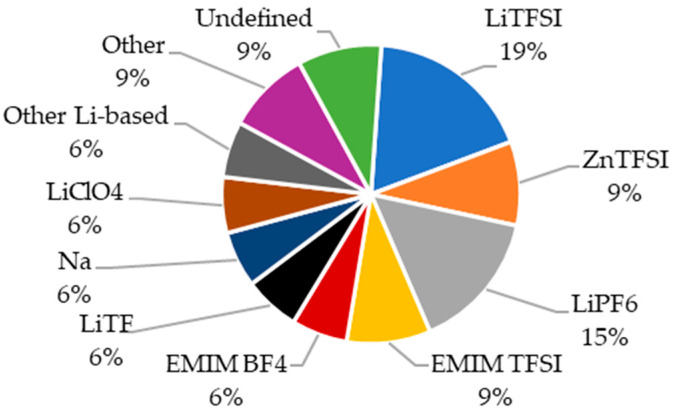
Proportional distribution of electrolyte types across the reviewed publications [7,8,42,45,47,53,55,56,57,58,59,60,61,62,63,64,65,66,67,68,69,70,71,72,73,74,75].

In terms of frequency in the reviewed publications (Figure 23), the most commonly used electrolytes in the production of multifunctional composite materials to date are those belonging to the LiTFSI group. Undefined electrolytes appeared in less than 10% of the articles, indicating the significant importance of this component, which researchers seem to recognize. The wide diversity of materials reflects ongoing efforts to identify an optimal solution.

**Figure 24:**
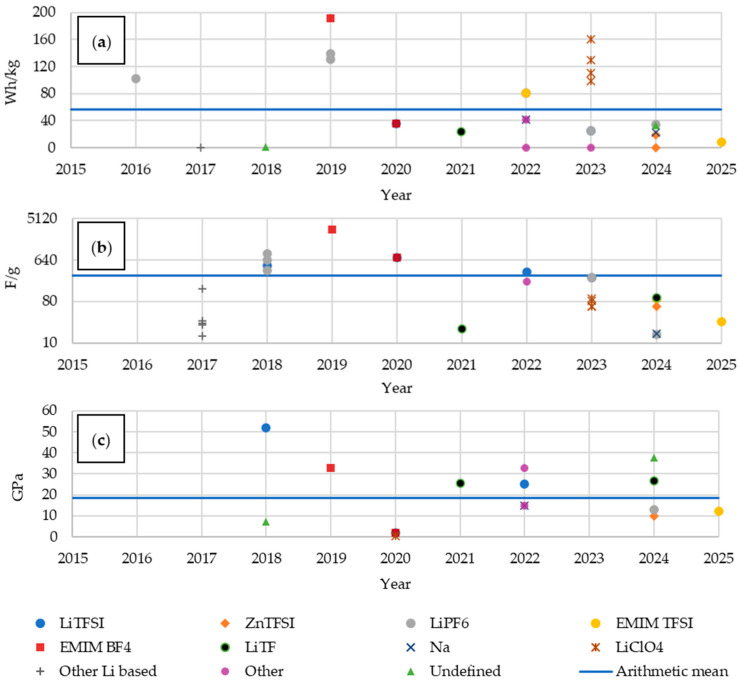
Electrochemical and mechanical properties over successive years for the considered electrolyte types: (**a**) Mass energy density, (**b**) Specific capacitance, (**c**) Electric strength [7,8,42,45,47,53,55,56,57,58,59,60,61,62,63,64,65,67,69,70,71,72,73,74,75].

The primary objective behind the use of various electrolytes is the enhancement of electrical performance. Based on the reviewed literature, it is possible to analyze both the gravimetric energy density (Figure 24a) and the specific capacity (Figure 24b). Multifunctional composites employing electrolytes based on LiClO_4_, LiPF_6_, and EMIM BF_4_ demonstrated results close to or above the average values across all the analyzed materials. For EMIM TFSI, both above-average and significantly low performance was observed. The remaining electrolytes yielded values below the average, with LiTf and ZnTFSI performing the worst.

The use of an EMIM BF_4_-based electrolyte yielded materials with the highest specific capacitance. LiTFSI and LiPF_6_ also enabled the development of energy-storing materials with typically above-average specific capacities. Among others, composites utilizing LiClO_4_ gave values close to the average capacity. LiTf, EMIM TFSI, and Na-based materials showed the poorest performance in this regard. The use of LiPF_6_-based electrolytes produced varied results, with some exceeding and others falling far below the average.

In terms of Young’s modulus (Figure 24c), the best-performing materials employed lithium-based electrolytes categorized under the “other” group [62]. Among the remaining publications, composites with LiTFSI and EMIM BF_4_ exhibited both very high and very low values. The use of LiClO_4_ resulted in the lowest mechanical performance, while the other electrolytes mostly clustered around the average.

### 3.6. Overview of Manufacturing Process Parameters

**Figure 25:**
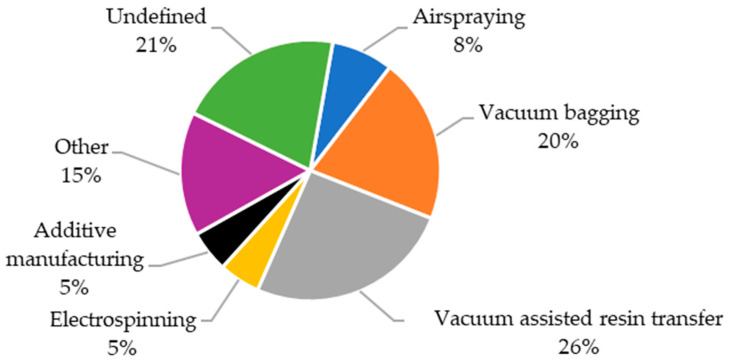
Proportional distribution of production methods across the reviewed publications [5,7,8,22,23,24,25,26,27,31,42,44,45,47,52,53,54,55,56,57,58,59,60,61,62,63,64,65,66,67,68,69,70,71,72,73,74,75,76].

In the reviewed publications (Figure 25), the most frequently reported manufacturing method was Vacuum Assisted Resin Transfer, followed by vacuum bagging. Other production techniques were significantly less popular, with none appearing in more than 10% of the articles where the manufacturing method was specified. It is also worth noting that over 20% of the reviewed papers did not clearly define the composite fabrication process. The chosen manufacturing method is closely related to the type of composite and the materials used.

**Figure 26:**
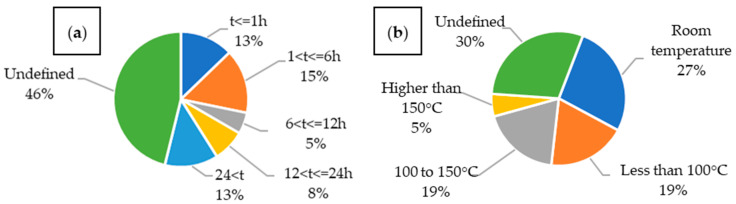
Proportional distribution of production parameters in publications (**a**) Curing time t; (**b**) Curing temperature [5,7,8,22,23,24,25,26,27,31,42,44,45,47,52,53,54,55,56,57,58,59,60,61,62,63,64,65,66,67,68,69,70,71,72,73,74,75,76].

Due to the diverse approaches taken by authors regarding composite curing, the process duration values (Figure 26a) were divided into five intervals, and the curing temperatures into four categories (Figure 26b). In the case of multistep curing procedures, the total duration was calculated as the sum of all the steps, while the maximum temperature reached during the process was taken as the representative value. In both cases, a significant proportion of publications did not clearly define the curing parameters. Based on the available data, it can be concluded that the most common curing conditions involved a total time of less than 6 h and a temperature below 100 °C. While curing conditions are typically specified by resin manufacturers, in the case of multifunctional composites, the curing temperature also affects the electrolyte. This is particularly important for composites incorporating LiPo batteries, where excessively high temperatures can lead to battery degradation.

## 4. Comparison of Described Multifunctional Composites for Electrical Energy Storage

In this section, the focus is placed on analyzing the mechanical and physical properties relevant to the design and application of composite materials in modern technologies. In Section 4.1, Section 4.2 and Section 4.3, the materials are compared based on three selected parameters. Subsequently, Section 4.4 highlights the best-performing solutions and provides a detailed characterization of each.

### 4.1. Energy Density—Tensile Strength—Young’s Modulus

The mechanical property of tensile strength was included since many composite structures operate under tensile loads, both in static and dynamic conditions. Energy density refers to the material’s ability to store energy, which is particularly important in composites designed to integrate mechanical and electrochemical functions. For example, a low mass combined with high energy storage capacity is a key parameter in applications such as aerospace, automotive, and mobile technologies.

**Figure 27:**
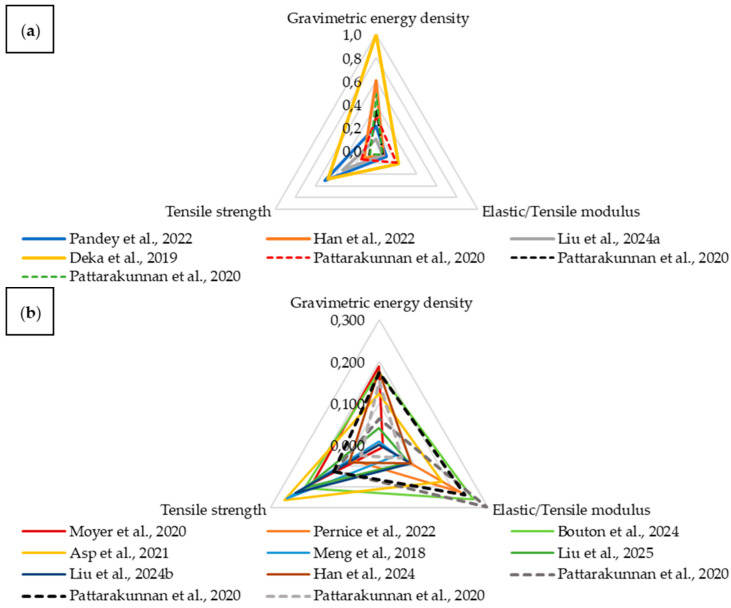
Normalized parameters with values in the range of: (**a**) 0.3 to 1.0, (**b**) below 0.3. Solid lines correspond to structural batteries, while dashed correspond to embedded LiPo batteries [8,23,42,54,57,58,59,60,64,65,67,71,75].

Following the described methodology, the material parameters were normalized and then plotted on a radar chart (Figure 27a). To improve readability, values below 0.3 were separated (Figure 27b). This group represents composites that performed significantly worse than others in terms of the analyzed properties. Additionally, dashed lines denote composites with embedded LiPo batteries, while solid lines correspond to structural batteries.

The charts show that structural batteries place greater emphasis on tensile strength, whereas Young’s modulus predominates in composites with embedded LiPo batteries. Subsequently, the area covered by each material relative to the total area of the chart was calculated (Table 1).

Structural batteries cover a larger area on the chart, indicating that they perform better as multifunctional composites compared to composites with embedded LiPo batteries based on the selected parameters. The greatest chart coverage was achieved by a composite using aramid fiber as the separator instead of glass fiber, as in most cases, and EMIM BF_4_ as the electrolyte.

### 4.2. Energy Density—Flexural Strength—Flexural Modulus

The next parameter analyzed is flexural strength, which, alongside tensile strength, is one of the most commonly encountered types of loading in structural applications. In many real-world structural systems, composite materials operate under bending loads caused by bending moments, making this property essential for assessing their suitability in the design of load-bearing and protective components. As with tensile strength, flexural strength was compared with energy density, allowing for an evaluation of the trade-off between the material’s ability to carry mechanical loads and its potential for energy storage while maintaining low overall weight.

**Figure 28:**
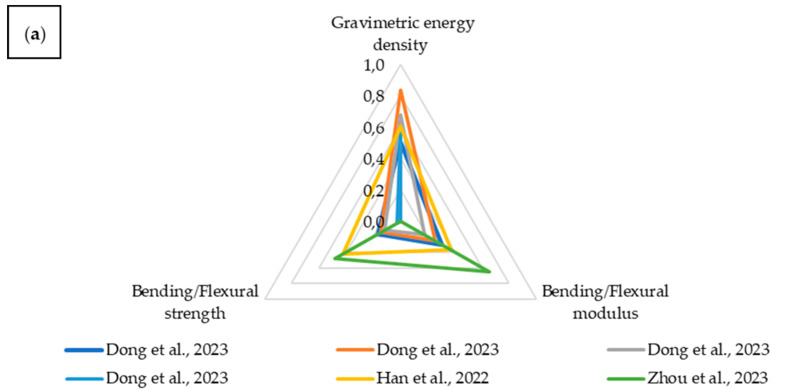

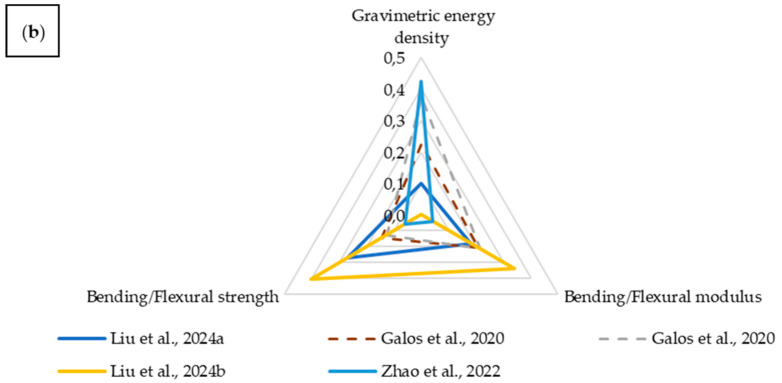
Normalized parameters with values in the range of: (**a**) 0.5 to 1.0, (**b**) below 0.5. Solid lines correspond to structural batteries, while dashed correspond to embedded LiPo batteries. [47,54,65,67,72,73,76].

The charts were again divided into two sections. Due to the range of normalized values, one includes those above 0.5 (Figure 28a), and the other those below 0.5 (Figure 28b). Composites with embedded LiPo batteries are marked with a dashed line.

In this case, the superiority of structural batteries over embedded LiPo batteries is again evident. For bending, the distinction between modulus and strength is not as pronounced, but energy density clearly dominates. One material stands out with significantly better mechanical properties in terms of flexural performance, though its energy storage capability is negligible. The chart areas occupied by each material are summarized in Table 2.

As in the previous case, structural batteries cover a larger area on the chart, indicating their superior performance compared to composites with embedded LiPo batteries in terms of flexural strength. Among the analyzed materials, when considering both flexural modulus and strength along with energy density, there are more composites that demonstrate comparably good performance than in the case of parameters related to tensile loading.

### 4.3. Specific Capacity—Tensile Strength—Young’s Modulus

Another key electrochemical parameter, alongside energy density, is specific capacity. This property refers to the amount of charge a material can store per unit mass (or volume), thus determining how much energy can be delivered at a given operating voltage. It is a crucial parameter when designing composites that serve both structural and energy storage functions, where maintaining a balance between storage capacity and mechanical properties is essential. In this analysis, the specific capacity was compared with the Young’s modulus and tensile strength to evaluate the relationship between stiffness, strength, and the electrochemical functionality of the material.

**Figure 29:**
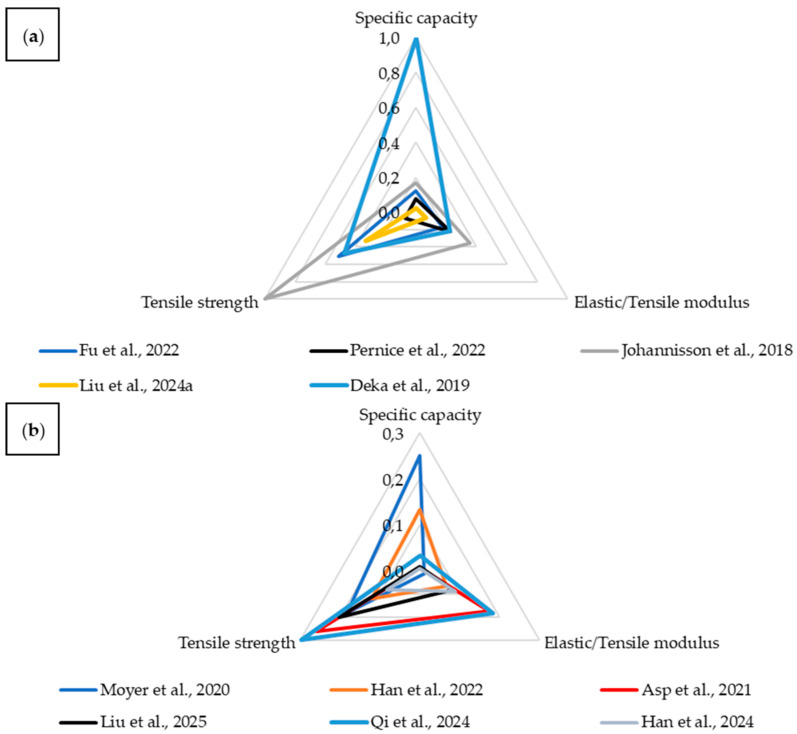
Normalized parameters of structural batteries with values in the range of: (**a**) 0.3 to 1.0, (**b**) below 0.3 [42,54,55,57,59,63,64,65,70,71,75].

In this comparison, no embedded battery composites were included, as none of them had all three parameters reported. A threshold value of 0.3 was used to separate high (Figure 29a) and low (Figure 29b) normalized values.

The analysis indicates that, in the context of specific capacity, more favorable relationships are observed with tensile strength than with Young’s modulus. This suggests that it is possible to achieve materials with significant mechanical strength while maintaining high energy storage capability, even if their stiffness remains at a moderate level. The area covered by each material on the plot is summarized in Table 3. When considering specific capacity, one material clearly stands out from the rest as a multifunctional composite.

### 4.4. Best Solutions

**Figure 30:**
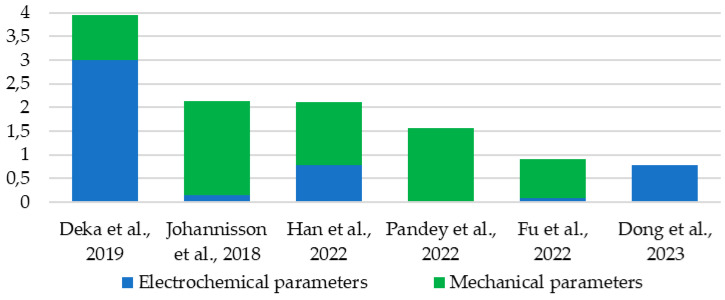
Sum of normalized parameters [8,47,54,55,63,75].

Of the comparisons presented, six materials were selected—two from each set—and their electrical and mechanical properties were compared through normalization. The normalized values for each parameter were summed separately for electrical and mechanical properties (Figure 30). This summed value allows the determination of which material performs better relative to the others. It can be observed that the composites described in [63,75] exhibit the best parameters as multifunctional materials, with one offering superior electrical performance, while the other shows the best mechanical strength characteristics but with poor electrical performance. It is worth noting that both of these materials are older designs, from 2019 and 2018, respectively. More recent materials, such as one described in [54], offer a better balance between mechanical and electrical properties.

#### 4.4.1. Parameters of Best Analyzed Materials

The analyzed materials were then examined for all the parameters studied and reported by the authors, divided into mechanical (Table 4) and electrical (Table 5) categories. A higher color saturation indicates a greater parameter value. The order of entries in the tables corresponds to the total sum of the normalized parameters (best on top).

#### 4.4.2. Detailed Characterization of Selected Composites

All the selected materials are in the form of laminates built using carbon fibers.

- In [63], T800 type fibers were used, and Bisphenol A served as the electrolyte, although detailed information about the resin, electrodes, or separator materials was not provided.
- In [75], T300 3K fiber combined with LSP-8020B resin was used. A distinctive feature of this material is the use of K49 aramid fiber as the separator, which likely contributed significantly to better mechanical properties compared to composites with glass fiber separators. The electrodes were made from a combination of carbon fiber, copper, and cobalt. The electrolyte used was the ionic compound EMIM BF_4_, and copper was used as the current collector.
- The authors in [54] also used T300 carbon fiber, but with an epoxy resin (GCC135) as the matrix. Carbon fiber served as the separator, while the electrodes consisted of MnOx/N-C nanotubes. The electrolyte was an aqueous solution of 2M ZnSO_4_ + 0.1M MnSO_4_ salt.
- Carbon fiber with graphene platelets was used in the composite described in [8], together with glass fiber as the separator. The anode was made of molybdenum dioxide (MoO_2_), and the cathode of manganese oxide (Mn_3_O_4_). A gel electrolyte based on 1M Na_2_SO_4_ was applied.
- A prepreg carbon fabric composite was combined with thin copper and aluminum plates [55]. The cured polymer electrolyte was prepared from PEGDA and E2BADMA and contained 1M LiTFSI dissolved in an EC/PC mixture, crosslinked using AIBN.
- T800 6K fiber was also used in another material produced by the drop-on-demand method [47]. In this case, the resin and separator were formed by a Loctite 3D 3955 prepreg. A polymer electrolyte consisting of a resin with a mixture of EC and PC solvents and a 1 molar solution of LiClO_4_, providing ionic conductivity, were used. The cathode consisted of carbon fiber coated with lithium phosphate enriched with conductive additives: carbon black (CB) and graphene. The current collector was made of copper and aluminum plates.

#### 4.4.3. Production Methods of Selected Composites

For producing high-quality multifunctional composite materials for energy storage, VARTM methods [54,75] and vacuum forming [55,63] have been applied. The curing times and temperatures are important factors when employing such techniques. Both room temperature curing [54] and elevated temperatures of 60 °C for 12 h [63] or 48 h [75] have been employed, as well as a two-step curing cycle of 90 °C for 0.5 h followed by 125 °C for 1.5 h [55]. The drop-on-demand method involved a layer thickness of 0.4 mm, with fibers first being soaked in acetone for 8 h and then left overnight in chloroform. Prior to production, the fibers were dried at 80 °C for 8 h [47].

## 5. Numerical Simulations

In the case of structural batteries or other composites performing additional functions (e.g., energy storage), we are dealing with a complex system resulting from the intricate material structure as well as the simultaneous mechanical and electrochemical interactions. Simulations allow a material’s behavior to be predicted before its physical fabrication, saving time and money, and helping to avoid costly mistakes. Besides mechanical parameters typical for composites, electrochemical simulations specific to batteries must also be conducted. The material properties are also influenced by the heating of the battery due to current flow, so thermal properties such as thermal conductivity must be anticipated. These phenomena occur simultaneously and mutually influence each other, which can be predicted by performing multiphysics analysis. This entails high model complexity and, consequently, significant computational power requirements. Proper execution of analyses requires knowledge of the material parameters. The conducted literature review indicates substantial gaps in data for individual materials, which complicates or even prevents comprehensive simulation of such materials in a given application. For composites, it is also crucial to consider an appropriate failure criterion.

In published studies, researchers have used various simulation methods, including the Finite Element Method (FEM), for mechanical and thermal analyses. To date, multifunctional composite materials have been most often modeled as a segment containing individual layers, which are then analyzed using FEM. This approach does not address a material’s behavior at its edges.

To accurately model the mechanical, thermal, and electrochemical processes occurring in a structural battery, coupled multiphysics models are necessary. During battery operation, the concentration of lithium ions changes, affecting the volume and mechanical properties of the electrodes (e.g., carbon fibers). Conversely, deformations and stresses can lead to cracking and disturbances in electrochemical processes. The entire system also generates heat, and the temperature distribution influences both the mechanical and electrical performance [37].

Using numerical methods, the following were determined:

- Stresses resulting from pressure during lamination and bending [33]
- Influence of the placement of LiPo batteries in composites on modal frequency and vibration damping [22]
- Impact of the number of batteries on composite behavior under tension and compression [23,31]
- Changes in composite temperature during discharge cycles with varying C-rates. The battery was omitted in the analyses. Behavior inside a satellite due to heating during discharge cycles [43,52]
- Behavior of LiPo batteries under high-speed impact based on low-speed impact data [30]
- Stiffness of structural batteries [79]
- Numerical models of structural batteries [47]
- Mechanical properties, including fatigue strength of structural composites [13]
- Behavior of honeycomb cores under compression, tension, and shear [88]
- Strength of sandwich panels with honeycomb cores under bending, compression, and resistance to high- and low-velocity impacts [89,90,91]

Fatigue simulations form a separate category. While fatigue strength is commonly studied in traditional composites [92], quantitative results for electrical energy storage multifunctional composites are practically nonexistent in the literature. Moreover, the underlying damage mechanisms remain largely unexplained. Nevertheless, this is a crucial aspect due to the practical applications of such materials. Besides fatigue resulting from mechanical loads, fatigue caused by cyclic electrical stresses and temperature fluctuations must also be taken into account.

## 6. Practical and Potential Applications of Multifunctional Materials

**Figure 31:**
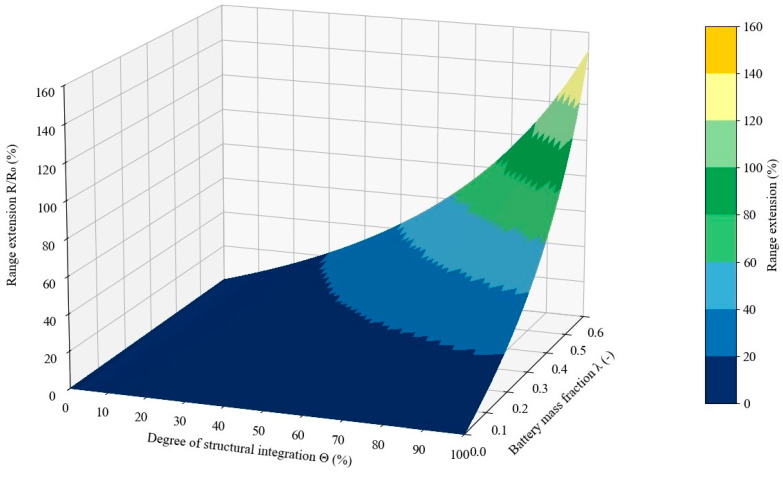
Theoretical impact of using a multifunctional composite on aircraft range during the cruise phase.

One of the fundamental advantages of high multifunctional efficiency is the reduction in system weight. In aerospace applications, integrating part of the battery mass into the structural component results in an increased flight range, and the results of our own calculations (Figure 31) are consistent with the analyses of other researchers [93]. This is a particularly attractive area of application due to the very high proportion of composite materials in aircraft, which exceeds 50% of the total weight [4]. The integration of structural batteries has been analyzed in the case of an unmanned fixed-wing aircraft. Replacing the conventional battery allowed for an approximately 10% increase in flight time [39]. A multifunctional composite storing energy has also been used as the casing of a drone to support the main power source of a quadcopter-type UAV [94]. Sandwich-type composite materials and structural batteries have been analyzed for satellite applications. FEM simulations have suggested that this is a promising solution, and tests of such applications have also been conducted [14,52].

Besides the aerospace industry, structural batteries are also an attractive solution for the automotive sector. The use of such composites has been analyzed in electric vehicles. In the case of the electric BMW i3, it is possible to increase the driving range by 70% while maintaining the original weight [95]. It has also been shown that replacing a passenger car’s roof with a structural battery could eliminate the conventional battery and reduce the total mass by 20% compared to a classic roof and battery. However, it is important to consider the high load on the battery during vehicle startup [3]. Structural batteries have been used in a toy car and combined with a photovoltaic panel in an IoT device [8].

## 7. Research Gap and Necessary Paths for Further Development of Multifunctional Materials

Fatigue studies of multifunctional composites are very limited, despite the fact that understanding damage mechanisms is crucial for their industrial applications. In the few studies conducted, a significant decrease in capacity was observed, while the initial internal resistance remained stable [24,31]. There is a need to critically recognize the limitations and challenges inherent in these materials, including mechanical-electrochemical trade-offs, degradation pathways, cost, and practical integration feasibility. There is an insufficient number of comprehensive analyses considering the interaction of mechanical and electrochemical processes, which hinders the prediction of the durability and reliability of these materials. Further experimental research and modeling are needed to fully assess their behavior under fatigue loading. The mechanisms of fatigue damage initiation and propagation in multifunctional materials remain poorly understood. There is a lack of detailed studies explaining the processes of microdamage formation under cyclic loading, which poses a serious barrier to the safe and reliable practical use of such systems.

The literature lacks information on in situ tests that would enable observation and analysis of multifunctional materials’ behavior under simultaneous real operational conditions, such as mechanical, thermal, and electrochemical loads in specific applications –for example, as a structural element of a UAV. Such tests are essential to fully understand damage and fatigue degradation mechanisms in these advanced materials.

Currently, there is no clearly defined development path for material selection in multifunctional structural batteries. The literature reveals a great diversity of fibers, matrices, and electrolytes used. Additionally, the absence of standardized methodologies limits reliable comparison and benchmarking of materials, complicating the evaluation of trade-offs between multifunctional properties. Each research group applies its own procedures, complicating the evaluation and compilation of data regarding mechanical properties, electrochemical properties, and durability. Standardization of research protocols and guidelines for the most promising composite components are therefore critical to accelerate progress in this field.

Multifunctional materials storing electrical energy can be further expanded by implementing additional functions that significantly broaden their applications. This may include energy harvesting, which would contribute to longer device operating times and promote the use of renewable energy sources. Moreover, the integration of structural health monitoring systems increases reliability in various applications. Equally important are thermal monitoring and management systems, due to battery temperature rise during operation. Careful design considering these additional functionalities is essential to balance performance, durability, and practical applicability.

An important issue requiring development is production methods that allow scalability and ensure adequate adhesion. Structural batteries demand increased mechanical strength and energy density. When drawing higher current, thermal management systems may be necessary.

Further work is required to understand the properties and production possibilities of multifunctional composites using solid electrolytes. Future research should also focus on combining more than two functions. An excellent example is integrating batteries with photovoltaic panels and piezoelectrics. One of the essential steps toward practical application of such materials is optimization of shape and layout, which is, however, specific to each application.

Models linking individual functionalities and their mutual interactions also need further development. Proper modeling requires conducting appropriate laboratory tests as well as tests in real environments where all factors act simultaneously. This integrated approach should allow the identification of critical material properties and interactions, supporting the effective application of multifunctional composites in real-world conditions.

## 8. Conclusions

This article presents a review of the current state of research on multifunctional composite materials, with particular emphasis on energy storage and the associated technological and research challenges.

Based on the reviewed literature, structural batteries currently represent the most promising solution. This approach allows significant tailoring of material parameters, achieving performance surpassing other types of integration, which makes it the most extensively studied method.

Using the adopted material evaluation method, a ranking was developed to identify those offering the best properties, considering all key parameters. Determining multifunctional efficiency for most materials is not possible due to the lack of suitable non-multifunctional reference material.

Trend analysis over the past decade shows that improvements in electrical performance have progressed faster than capacity decline, which parallels developments in mechanical properties. Initially, mechanical properties were high but were subsequently optimized to balance mechanical and electrochemical parameters and durability.

Examining individual parameters in relation to composite constituents reveals the dependence of electrical parameters on materials responsible for structural functions and vice versa. This relationship is crucial for the selection and design of new materials and components. A clear tendency to improve electrochemical properties at the expense of mechanical properties is also observed.

So far, structural batteries have been implemented in several demonstrators, confirming the validity of this concept. Practical applications have mainly been analyzed in the aerospace, space, and automotive industries. Integration possibilities have been supported by a limited number of simulations. Additionally, the potential for integrating extra functions, such as energy generation, via photovoltaic panels or piezoelectric materials has been explored.

Despite progress, a long way remains to reach the successful implementation of multifunctional composites for energy storage. Further development of this promising field requires comprehensive fatigue and in situ studies of multifunctional materials, clear guidelines for material selection, and standardized research methodologies. The advancement of models combining multiple functionalities, based on extensive laboratory and field testing, is also essential.

## Figures and Tables

**Table 1:** Percentage of chart area occupied by each material corresponding to Figure 27.

Type	Structural Battery											
Source	[8]	[42]	[54]	[57]	[58]	[59]	[60]	[64]	[65]	[67]	[71]	[75]
Area [%]	6.1	1.3	4.0	0.6	4.5	3.3	0.5	0.9	2.1	0.7	1.3	26.9
**Type**	**Embedded LiPo**											
Source	[23]	[23]	[23]	[23]	[23]	[23]						
Area [%]	2.1	3.1	4.4	0.7	1.6	2.1						

**Table 2:** Percentage of chart area occupied by each material corresponding to Figure 28.

Type	Structural Battery									Embedded LiPo	
Source	[47]	[47]	[47]	[47]	[54]	[65]	[67]	[72]	[73]	[76]	[76]
Area [%]	9.7	12.3	7.4	0.5	21.2	3.1	4.6	10.5	1.5	3.6	5.1

**Table 3:** Percentage of chart area occupied by each material corresponding to Figure 29.

Type	Structural Battery										
Source	[42]	[54]	[55]	[57]	[59]	[63]	[64]	[65]	[70]	[71]	[75]
Area [%]	1.7	1.1	5.7	1.3	1.6	19.5	0.7	1.0	2.3	0.3	26.9

**Table 4:** Mechanical properties of selected multifunctional composites. Darker colors indicate higher values of the respective parameter.

Source	Tensile Modulus [GPa]	Flexural Modulus [GPa]	Shear Modulus [GPa]	Tensile Strength [Mpa]	Compressive Strength [Mpa]	Flexural Strength [Mpa]	Shear Strength [Mpa]	Impact Resistance [J]	Impact Strength [J/m]
[75]	32.65			488.89					
[63]	52		1.5	982	997		13.2		
[54]	10	12.1		154.3		203.2			
[8]	15			518		477			2666
[55]	25.3			523				30	
[47]		8.5				70			

**Table 5:** Electrochemical and electronic properties of selected multifunctional composites. Darker colors indicate higher values of the respective parameter.

Source	Gravimetric Energy Density [Wh/kg]	Volumetric Energy Density [Wh/l]	Specific Capacity [F/g]	Cycle Life at 0.1C
[75]	191.64		2863	2000
[63]			200	
[54]	115.2		85	500
[8]	41.4	10.33		
[55]			143	150
[47]	160		90	

## Data Availability

No new data were created or analyzed in this study. Data sharing is not applicable to this article.

## References

[1] Zhao Y., Pohl O., Bhatt A.I., Collis G.E., Mahon P.J., Rüther T., Hollenkamp A.F. (2021). A Review on Battery Market Trends, Second-Life Reuse, and Recycling. Sustain. Chem..

[2] Yang Z., Mu Y., Acauan L.H., Fang J.H., Rogers M., Majeed M.K., Zhou Y. (2024). Understanding and Recent Advances on Lithium Structural Batteries. Chem. Eng. J..

[3] Jin T., Singer G., Liang K., Yang Y. (2023). Structural Batteries: Advances, Challenges and Perspectives. Mater. Today.

[4] Skoczylas J., Samborski S., Kłonica M. (2019). The Application of Composite Materials in the Aerospace Industry. J. Technol. Exploit. Mech. Eng..

[5] Morel C., Baranger E., Lamon J., Braun J., Lorrette C. Understanding the influence of manufacturing defects on the tensile behavior of sic/sic filament wound tubes from a unidirectional composite model. Proceedings of the 20th European Conference on Composite Materials.

[6] Zhu J., Wierzbicki T., Li W. (2018). A Review of Safety-Focused Mechanical Modeling of Commercial Lithium-Ion Batteries. J. Power Sources.

[7] Ladpli P., Nardari R., Kopsaftopoulos F., Chang F.K. (2019). Multifunctional Energy Storage Composite Structures with Embedded Lithium-Ion Batteries. J. Power Sources.

[8] Pandey D., Sambath Kumar K., Henderson L.N., Suarez G., Vega P., Salvador H.R., Thomas J. (2022). Energized Composites for Electric Vehicles: A Dual Function Energy-Storing Supercapacitor-Based Carbon Fiber Composite for the Body Panels. Small.

[9] Kühnelt H., Beutl A., Mastropierro F., Laurin F., Willrodt S., Bismarck A., Romano F. (2021). Structural Batteries for Aeronautic Applications—State of the Art, Research Gaps and Technology Development Needs. Aerospace.

[10] Al-Furjan M.S.H., Shan L., Shen X., Zarei M.S., Hajmohammad M.H., Kolahchi R. (2022). A Review on Fabrication Techniques and Tensile Properties of Glass, Carbon, and Kevlar Fiber Reinforced Polymer Composites. J. Mater. Res. Technol..

[11] Chiu L.N., Falzon B.G., Boman R., Chen B., Yan W. (2015). Finite element modelling of composite structures under crushing load. Compos. Struct..

[12] Zhao Y.Q., Zhou Y., Huang Z.M., Batra R.C. (2019). Experimental and micromechanical investigation of T300/7901 unidirectional composite strength. Polym. Compos..

[13] Zhang Y., Zheng T., Liu G., Lu H., Li G., Zong Q., Zhang W. (2025). Predicting the fatigue life of T800 carbon fiber composite structural component based on fatigue experiments of unidirectional laminates. Int. J. Fatigue.

[14] Prashanth S., Subbaya K.M., Nithin K., Sachhidananda S. (2017). Fiber reinforced composites—A review. J. Mater. Sci. Eng..

[15] Jagannatha T.D., Harish G. (2015). Mechanical properties of carbon/glass fiber reinforced epoxy hybrid polymer composites. Int. J. Mech. Eng. Robot. Res..

[16] Goh G.D., Dikshit V., Nagalingam A.P., Goh G.L., Agarwala S., Sing S.L., Yeong W.Y. (2018). Characterization of mechanical properties and fracture mode of additively manufactured carbon fiber and glass fiber reinforced thermoplastics. Mater. Des..

[17] Rajak D.K., Wagh P.H., Linul E. (2021). Manufacturing technologies of carbon/glass fiber-reinforced polymer composites and their properties: A review. Polymers.

[18] Dharmavarapu P., Sreekara Reddy M.B.S. (2022). Aramid fibre as potential reinforcement for polymer matrix composites: A review. Emerg. Mater..

[19] Rafique I., Kausar A., Muhammad B. (2016). Epoxy resin composite reinforced with carbon fiber and inorganic filler: Overview on preparation and properties. Polym.-Plast. Technol. Eng..

[20] Sairajan K.K., Aglietti G.S., Mani K.M. (2016). A review of multifunctional structure technology for aerospace applications. Acta Astronaut..

[21] Yoo S., Hong C., Chong K.T., Seul N. (2019). Analysis of pouch performance to ensure impact safety of lithium-ion battery. Energies.

[22] Galos J., Khatibi A.A., Mouritz A.P. (2019). Vibration and acoustic properties of composites with embedded lithium-ion polymer batteries. Compos. Struct..

[23] Pattarakunnan K., Galos J., Das R., Mouritz A.P. (2020). Tensile properties of multifunctional composites embedded with lithium-ion polymer batteries. Compos. Part A Appl. Sci. Manuf..

[24] Pattarakunnan K., Galos J., Das R., Mouritz A.P. (2021). Impact damage tolerance of energy storage composite structures containing lithium-ion polymer batteries. Compos. Struct..

[25] Zhang L., Lu S., Wang X., Ma K., Liu H., Zhou L. (2019). Manufacture and mechanical properties of sandwich structure-battery composites. J. Polym. Eng..

[26] Galos J., Fredriksson C., Das R. (2021). Multifunctional sandwich panel design with lithium-ion polymer batteries. J. Sandw. Struct. Mater..

[27] Biswas P.K., Liyanage A.A.H., Jadhav M., Agarwal M., Dalir H. (2022). Higher strength carbon fiber lithium-ion polymer battery embedded multifunctional composites for structural applications. Polym. Compos..

[28] Placke T., Kloepsch R., Dühnen S., Winter M. (2017). Lithium ion, lithium metal, and alternative rechargeable battery technologies: The odyssey for high energy density. J. Solid State Electrochem..

[29] Kisters T., Sahraei E., Wierzbicki T. (2017). Dynamic impact tests on lithium-ion cells. Int. J. Impact Eng..

[30] Kermani G., Sahraei E. (2019). Dynamic impact response of lithium-ion batteries, constitutive properties and failure model. RSC Adv..

[31] Attar P., Galos J., Best A.S., Mouritz A.P. (2020). Compression properties of multifunctional composite structures with embedded lithium-ion polymer batteries. Compos. Struct..

[32] Liang X., Tan F., Wei F., Du J. (2019). Research progress of all solid-state thin film lithium battery. IOP Conf. Ser. Earth Environ. Sci..

[33] Kammoun M., Berg S., Ardebili H. (2015). Flexible thin-film battery based on graphene-oxide embedded in solid polymer electrolyte. Nanoscale.

[34] Xia Q., Zan F., Zhang Q., Liu W., Li Q., He Y., Xia H. (2023). All-solid-state thin film lithium/lithium-ion microbatteries for powering the Internet of things. Adv. Mater..

[35] Strauss F., Kitsche D., Ma Y., Teo J.H., Goonetilleke D., Janek J., Brezesinski T. (2021). Operando characterization techniques for all-solid-state lithium-ion batteries. Adv. Energy Sustain. Res..

[36] Wu B., Chen C., Danilov D.L., Eichel R.A., Notten P.H. (2023). All-solid-state thin film Li-ion batteries: New challenges, new materials, and new designs. Batteries.

[37] Asp L.E., Johansson M., Lindbergh G., Xu J., Zenkert D. (2019). Structural battery composites: A review. Funct. Compos. Struct..

[38] Xu Y., Lu W., Xu G., Chou T.W. (2021). Structural supercapacitor composites: A review. Compos. Sci. Technol..

[39] Danzi F., Salgado R.M., Oliveira J.E., Arteiro A., Camanho P.P., Braga M.H. (2021). Structural batteries: A review. Molecules.

[40] Tsushima N., Su W. (2018). A study on adaptive vibration control and energy conversion of highly flexible multifunctional wings. Aerosp. Sci. Technol..

[41] Akbar M., Curiel-Sosa J.L. (2016). Piezoelectric energy harvester composite under dynamic bending with implementation to aircraft wingbox structure. Compos. Struct..

[42] Moyer K., Meng C., Marshall B., Assal O., Eaves J., Perez D., Pint C.L. (2020). Carbon fiber reinforced structural lithium-ion battery composite: Multifunctional power integration for CubeSats. Energy Storage Mater..

[43] Pattarakunnan K., Galos J., Das R., Best A.S., Kyratzis I.L., Mouritz A.P. (2023). Internal heating of energy storage composites containing lithium-ion polymer batteries. Compos. Part A Appl. Sci. Manuf..

[44] Li H., Zhou D., Cao J., Li Z., Zhang C. (2023). On the damage and performance degradation of multifunctional sandwich structure embedded with lithium-ion batteries under impact loading. J. Power Sources.

[45] Zhang Y., Ma J., Singh A.K., Cao L., Seo J., Rahn C.D., Hickner M.A. (2017). Multifunctional structural lithium-ion battery for electric vehicles. J. Intell. Mater. Syst. Struct..

[46] Shah M., Chaudhary V. (2020). Flow modeling and simulation study of vacuum assisted resin transfer molding (VARTM) process: A review. IOP Conf. Ser. Mater. Sci. Eng..

[47] Dong X., Chen Y. (2023). Multifunctional additive manufacturing and multiphysics numerical investigations of carbon fiber structural battery composite using a drop-on-demand method with in-situ consolidation. Mater. Des..

[48] (2018). Electrically Propelled Road Vehicles—Test Specification for Lithium-Ion Traction Battery Packs and Systems —Part 4: Performance Testing of Battery Packs and Systems.

[49] Finck D., Seidel C., Ostermeier A., Hausmann J., Rief T. (2020). Experimental investigation on the in-plane creep behavior of a carbon-fiber sheet molding compound at elevated temperature at different stress states. Materials.

[50] (1998). Fibre-Reinforced Plastic Composites—Determination of Flexural Properties.

[51] (2015). Standard Test Methods for Flexural Properties of Unreinforced and Reinforced Plastics and Electrical Insulating Materials.

[52] Wang Y., Peng C., Zhang W. (2015). Thermal analysis of multifunctional structural battery for satellite applications. Appl. Therm. Eng..

[53] Thakur A., Dong X. (2020). Printing with 3D continuous carbon fiber multifunctional composites via UV-assisted coextrusion deposition. Manuf. Lett..

[54] Han Q., Sheng Y., Liu X., Zhang X., Chen X., Li B., Han Z. (2022). Carbon fiber reinforced epoxy composite combining superior electrochemical energy storage and mechanical performance. Chem. Eng. J..

[55] Fu Y., Chen Y., Yu X., Zhou L. (2022). Fiber metal laminated structural batteries with multifunctional solid polymer electrolytes. Compos. Sci. Technol..

[56] Choi J.S., Park H.W., Noh J.E., Cha J., Jang W.H., Kim C.G. (2023). Composite-fabric-based structure-integrated energy storage system. Compos. Struct..

[57] Pernice M.F., Qi G., Senokos E., Anthony D.B., Nguyen S., Valkova M., Kucernak A.R. (2022). Mechanical, electrochemical and multifunctional performance of a CFRP/carbon aerogel structural supercapacitor and its corresponding monofunctional equivalents. Multifunct. Mater..

[58] Bouton K., Schneider L., Zenkert D., Lindbergh G. (2024). A structural battery with carbon fibre electrodes balancing multifunctional performance. Compos. Sci. Technol..

[59] Asp L.E., Bouton K., Carlstedt D., Duan S., Harnden R., Johannisson W., Zenkert D. (2021). A structural battery and its multifunctional performance. Adv. Energy Sustain. Res..

[60] Meng C., Muralidharan N., Teblum E., Moyer K.E., Nessim G.D., Pint C.L. (2018). Multifunctional structural ultrabattery composite. Nano Lett..

[61] Fredi G., Jeschke S., Boulaoued A., Wallenstein J., Rashidi M., Liu F., Asp L.E. (2018). Graphitic microstructure and performance of carbon fibre Li-ion structural battery electrodes. Multifunct. Mater..

[62] Yu Y., Zhang B., Feng M., Qi G., Tian F., Feng Q., Wang S. (2017). Multifunctional structural lithium ion batteries based on carbon fiber reinforced plastic composites. Compos. Sci. Technol..

[63] Johannisson W., Ihrner N., Zenkert D., Johansson M., Carlstedt D., Asp L.E., Sieland F. (2018). Multifunctional performance of a carbon fiber UD lamina electrode for structural batteries. Compos. Sci. Technol..

[64] Liu X., Peng Y., Zhou L. (2025). Coupled carbon fiber structural battery composites with reinforced interfaces to improve multifunctional performance. Chem. Eng. J..

[65] Liu X., Zhou L. (2024). Multifunctional composite electrolytes for mechanically-robust and high energy density carbon fiber structural batteries. Chem. Eng. J..

[66] Li J., Liu X., Hu Z., Liu Y., Li H., Zhou L. (2024). Interface reinforced by polymer binder for expandable carbon fiber structural lithium-ion battery composites. Compos. Sci. Technol..

[67] Liu X., Zhou L. (2024). Curved surface coupled structural battery composites manufactured by resin transfer molding process: Microstructure and multifunctional performance. Compos. Commun..

[68] Xun L., Li C., Meng Q., Wang Z., Guo Y., Zheng K., Zhao T. (2024). High-Strength and High-Temperature-Resistant Structural Battery Integrated Composites via Polymeric Bi-Continuous Electrolyte Engineering. Adv. Sci..

[69] Iyer V., Petersen J., Geier S., Wierach P. (2024). Development and multifunctional characterization of a structural sodium-ion battery using a high-tensile-strength poly(ethylene oxide)-based matrix composite. ACS Appl. Energy Mater..

[70] Qi G., Wu Y., Ding Y., Zhang B. (2024). Multifunctional performances of structural battery composite full-cells based on carbon fiber anode and LiFePO_4_ loaded carbon fiber cathode. Polym. Test..

[71] Han Z., Zhu J., Feng Y., Zhang W., Xiong Y., Zhang W. (2024). Manufacturing carbon fabric composite structural batteries using spray with high-pressure and high-temperature and vacuum-bag assisted infusion techniques. Compos. Sci. Technol..

[72] Zhou H., Su Y., Zhang J., Li H., Zhou L., Huang H. (2023). A novel embedded all-solid-state composite structural supercapacitor based on activated carbon fiber electrode and carbon fiber reinforced polymer matrix. Chem. Eng. J..

[73] Zhao Y., Xu H., Cai G., Yan C., Liu D., Chen G., Zhu Y. (2022). Multi-functional structural supercapacitor based on manganese oxide-hydroxide nanowires modified carbon fiber fabric electrodes. Polym. Compos..

[74] Kwon O., Deka B.K., Kim J., Park H.W. (2018). Electrochemical performance evaluation of tin oxide nanorod-embedded woven carbon fiber composite supercapacitor. Int. J. Energy Res..

[75] Deka B.K., Hazarika A., Kim J., Kim N., Jeong H.E., Park Y.B., Park H.W. (2019). Bimetallic copper cobalt selenide nanowire-anchored woven carbon fiber-based structural supercapacitors. Chem. Eng. J..

[76] Galos J., Best A.S., Mouritz A.P. (2020). Multifunctional sandwich composites containing embedded lithium-ion polymer batteries under bending loads. Mater. Des..

[77] Galos J., Pattarakunnan K., Best A.S., Kyratzis I.L., Wang C.H., Mouritz A.P. (2021). Energy storage structural composites with integrated lithium-ion batteries: A review. Adv. Mater. Technol..

[78] (2020). Standard Test Method for Core Shear Properties of Sandwich Constructions by Beam Flexure.

[79] Singh A.K., Cao L., Ma J., Seo J., Bakis C.E., Zhang Y., Rahn C.D. (2015). Design, manufacture and test of a novel structural battery based on sandwich construction. J. Sandw. Struct. Mater..

[80] Teijin Carbon Filament Yarn. https://www.teijincarbon.com/products/filament-yarn/.

[81] Saba N., Jawaid M., Alothman O.Y., Paridah M.T., Hassan A. (2016). Recent advances in epoxy resin, natural fiber-reinforced epoxy composites and their applications. J. Reinf. Plast. Compos..

[82] West System Inc 105 Epoxy System. https://www.westsystem.com/products/105-system/.

[83] Li Z., Wang L., Huang X., He X. (2024). Lithium bis(trifluoromethanesulfonyl)imide (LiTFSI): A prominent lithium salt in lithium-ion battery electrolytes–fundamentals, progress, and future perspectives. Adv. Funct. Mater..

[84] Liu Z., Xi M., Sheng R., Huang Y., Ding J., Tan Z., Wang Y. (2025). Zn(TFSI)_2_-mediated ring-opening polymerization for electrolyte engineering toward stable aqueous zinc metal batteries. Nano-Micro Lett..

[85] Wei Y., Chen W., Ge X., Liang J., Xing Z., Zhang Q., Wang Z.X. (2023). A flexible, highly conductive, tough ionogel electrolyte containing LiTFSI salt and ionic liquid [EMIM][TFSI] based on PVDF-HFP for high-performance supercapacitors. Polymer.

[86] Ray A., Saruhan B. (2021). Application of ionic liquids for batteries and supercapacitors. Materials.

[87] Geiculescu O.E., Stanga M., Navarrini W., Creager S.E., DesMarteau D.D. (2016). Solid polymer electrolytes from lithium (perfluorovinylether) sulfonate—Derived salts dissolved in high-molecular-weight poly(ethylene oxide). J. Fluor. Chem..

[88] Seemann R., Krause D. (2017). Numerical modelling of Nomex honeycomb sandwich cores at meso-scale level. Compos. Struct..

[89] Sun G., Huo X., Chen D., Li Q. (2017). Experimental and numerical study on honeycomb sandwich panels under bending and in-panel compression. Mater. Des..

[90] Sun G., Chen D., Wang H., Hazell P.J., Li Q. (2018). High-velocity impact behaviour of aluminium honeycomb sandwich panels with different structural configurations. Int. J. Impact Eng..

[91] Zhang X., Xu F., Zang Y., Feng W. (2020). Experimental and numerical investigation on damage behavior of honeycomb sandwich panel subjected to low-velocity impact. Compos. Struct..

[92] Ansari M.T.A., Singh K.K., Azam M.S. (2018). Fatigue damage analysis of fiber-reinforced polymer composites—A review. J. Reinf. Plast. Compos..

[93] Adam T.J., Liao G., Petersen J., Geier S., Finke B., Wierach P., Wiedemann M. (2018). Multifunctional composites for future energy storage in aerospace structures. Energies.

[94] Lim G.J.H., Chan K.K., Sutrisnoh N.A.A., Srinivasan M. (2022). Design of structural batteries: Carbon fibers and alternative form factors. Mater. Today Sustain..

[95] Carlstedt D., Asp L.E. (2020). Performance analysis framework for structural battery composites in electric vehicles. Compos. B Eng..

